# Calculation of *V*_S,max_ and Its Use as a Descriptor for the Theoretical Calculation of p*K*a Values for Carboxylic Acids

**DOI:** 10.3390/molecules24010079

**Published:** 2018-12-26

**Authors:** Guillermo Caballero-García, Gustavo Mondragón-Solórzano, Raúl Torres-Cadena, Marco Díaz-García, Jacinto Sandoval-Lira, Joaquín Barroso-Flores

**Affiliations:** Centro Conjunto de Investigación en Química Sustentable UAEM-UNAM, Unidad San Cayetano, Carretera Toluca–Atlacomulco km 14.5, Personal de la UNAM, Toluca 50200, Mexico; gcaballerog91@gmail.com (G.C.-G.); gustavms93@gmail.com (G.M.-S.); torres.cadena.raul@gmail.com (R.T.-C.); diaz.marco95@outlook.com (M.D.-G.); jsandovalira@gmail.com (J.S.-L.)

**Keywords:** p*K*a, hydrogen bond, maximum surface potential, σ–hole

## Abstract

The theoretical calculation of p*K*a values for Brønsted acids is a challenging task that involves sophisticated and time-consuming methods. Therefore, heuristic approaches are efficient and appealing methodologies to approximate these values. Herein, we used the maximum surface electrostatic potential (*V*_S,max_) on the acidic hydrogen atoms of carboxylic acids to describe the H-bond interaction with water (the same descriptor that is used to characterize σ-bonded complexes) and correlate the results with experimental p*K*a values to obtain a predictive model for other carboxylic acids. We benchmarked six different methods, all including an implicit solvation model (water): Five density functionals and the Møller–Plesset second order perturbation theory in combination with six different basis sets for a total of thirty-six levels of theory. The ωB97X-D/cc-pVDZ level of theory stood out as the best one for consistently reproducing the reported p*K*a values, with a predictive power of 98% correlation in a test set of ten other carboxylic acids.

## 1. Introduction

The hydrogen bond is a strong, directional, non-covalent interaction responsible for a large number of chemical phenomena spanning from chemistry to biochemistry [1,2,3], which has become a paradigm amongst the toolbox of chemical concepts [4,5]. Hydrogen bonding is also a major driving force of chemical reactivity. For instance, the deprotonation of Brønsted acids in aqueous media, where the reaction constant, *K*_a_, and its associated logarithmic quantity, p*K*a = −log*K*a, are an intrinsic characteristic of each acid. This process occurs through the abstraction of the acidic hydrogen atom using a water molecule (Equation 1). It is commonly regarded that the deprotonation reaction is mainly promoted by electrostatic interactions, given the partial positive charge on the acid hydrogen. Characterization of a protic acid through the p*K*a values is of practical importance and usefulness in various steps of the chemical design rationale, and therefore, it is an important quantity.

HA + H_2_O → H_3_O^+^ + A^−^ (1)

**Scheme 1:**
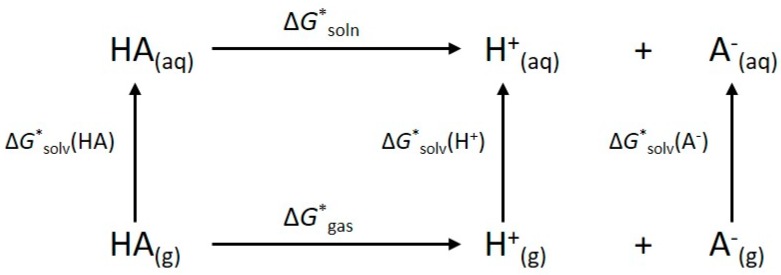
Thermodynamic cycle for a protic acid deprotonation.

The accurate prediction of p*K*a values for carboxylic acids by means of computational methods covers a wide range of potential applications from chemical design and biochemistry research to drug development [6,7,8]. However, calculating the equilibrium constant for the deprotonation reaction of a Brønsted acid implies the calculation of the Gibbs Free Energy change, (−Δ*G*), which in turn entails the calculation of extremely accurate solvation energies for all species involved (Scheme 1). Calculation of accurate solvation free energies remains challenging, since it requires the use of sophisticated and computationally intensive methods, such as G3MP2 or CBS-QB3 [9], mostly due to a poor description of the solute–solvent interactions [10]. This method is highly sensitive to even slight deviations, since an error of only 1.36 kcal/mol—Barely above chemical accuracy—Leads to a unit error in the p*K*a, making it impractical for large molecules.

Since the interaction of water molecules with the acidic hydrogen atom is not isotropic, some parallels between hydrogen and σ–hole bonded systems arise. The formation of these directional interactions implies the presence of an electrostatic potential maximum located on the opposite side of the O–H σ–bond, which can be quantified by the maximum surface electrostatic potential, *V*_S,max_. By assuming that the deprotonation of a carboxylic acid begins with the formation of a hydrogen bond with a water molecule, RCOOH···H_2_O, we propose the use of *V*_S,max_ as a suitable descriptor for the strength of this interaction, which in turn correlates with the corresponding p*K*a values, in a similar fashion to how a σ-hole-based interaction is quantified in halogen or tetrel bonds. Previously, the nucleophilicities and electrophilicities of Lewis acids and bases, respectively, have been derived from the interpolation of the mutual dissociation energies [11].

Previous efforts for deriving suitable p*K*a descriptors from ab initio or DFT descriptors have been successfully published, in some cases mixing implicit and explicit solvation models [12]. Electrostatic properties, such as the total molecular electrostatic potential (MEP) on the acidic hydrogen, combined with the sum of the valence Natural Atomic Orbital (NAO) energies on the acidic atom and the leaving proton for amino acids and nucleotides exhibits a correlation coefficient *R*^2^ = 0.91 [13] with the experimental p*K*a values. Monard and Jensen used various kinds of atomic charges of the conjugated phenolates, alkoxides, or thiolates, with the best correlations being observed for the atomic electrostatic charges from a Natural Population Analysis (NPA) calculated at the B3LYP/3-21G (*R*^2^ = 0.995) and M06-2X/6-311G (*R*^2^ = 0.986) levels of theory for alcohols and thiols, in implicit solvent, respectively. Other efforts include correlations on the excited states of photoacids [14] using Time Dependent DFT at the ωB97X-D/6-31G(*d*) level of theory for a family of hydroxyl-substituted aromatic compounds. QSPR models have yielded, for instance, a three parameters model which uses the MEP maxima, the number of carboxylic acid and amine groups for phenols, at the HF/6-31G(*d*,*p*) and B3LYP/6-31G(*d*,*p*) levels of theory (*R*^2^ = 0.96) [15]. It involves a four parameters linear equation comprising the highest normal mode vibrational frequency, the partial positive and negative charges divided by the total surface area and a reactivity index, defined in terms of a population analysis on the frontier orbital HOMO (*R*^2^ ca. 0.95 sic.) [16] for N-Base ligands at the semi empirical AM1 level of theory, as well as a Principal Components Analysis (PCA) for organic and inorganic acids (RMSE = 0.0195) [17]. Moreover, genetic algorithms (GA) and neural networks (NN) have employed frontier orbital energies for a chemical space of sixty commercial drugs [18] (GA, *R*^2^ = 0.703; NN, *R*^2^ = 0.929). Thus far, the only major commercial program capable of including the effects of molecular conformations on the estimation of p*K*a values is ‘Jaguar p*Ka*′ [19,20]. For more thorough reviews on the development of p*K*a descriptors, please refer to References [21,22,23].

Non-covalent interactions like tetrel [24], pnicogen [25], chalcogen [26,27], carbon [28], and halogen [29,30,31] bonds offer some resemblances to H-bonded systems, both in structural and reactivity terms. All these forms of bonding correspond to directional, intermolecular non-covalent interactions of an electrostatic nature involving elements in groups 14 through 17, respectively. These atoms behave as electrophiles through the interaction with either *n* or π electrons from Lewis bases [32,33]. The formation of these non-covalent interactions stems from a similar origin, via the presence of σ–holes [34,35,36], a localized region of positive electrostatic potential on the surface of the bridge atom (prominently present in atoms of group 17), and opposite to the internuclear axis of one of the covalent σ bonds, hence the σ–holes. A stretch of this label has been applied to hydrogen bonding, despite the absence of *p* electrons on hydrogen atoms and the high polarizability of the hydrogen bonds [37,38,39].

Energetically, the strength of these interactions increases as the bridging atom increases its atomic number, the electronegativity of the atom bonded opposite to the non-covalent bond, and the number of electron-withdrawing groups bonded to the bridging atom. Tetrel bonds, for instance, are stabilizing interactions in nature [40,41] that form cooperative networks [26,42,43,44,45,46], a feature that is used as a powerful tool for the design of crystal structures [47,48,49]. The stabilization arising from these interactions ranges from 1 kcal/mol to 50 kcal/mol [50]. Therefore, the formation and strength of these interactions closely depends on the polarization of the electron density surrounding the bridging atom. In the particular case of tetrel bonds, these factors have been extensively investigated by Scheiner, who has further assessed the electronic [50,51] and steric [52] contributions.

Several computational studies on the nature of tetrel bonds have been published so far, from their strict quantum treatment [53] to their charge transfer dynamics in the attoseconds regime [54], and the tunneling bond-breaking processes promoted by σ-holes [55].

Thus, the importance of the study of non-covalent interactions has large implications for crystal engineering [56], biochemistry, and the understanding of chemical reactivity [57,58,59]. In our research group, we have reported the chemical reduction of a trichloromethyl group into a methyl group via the attack of σ–holes on chlorine atoms by thiophenolate anions, a reaction mechanism which is extensible to other trichloromethyl compounds [60].

Herein, we presented a benchmark of linear models which correlate the *V*_S,max_ calculations with various DFT methods, and used MP2 as a reference (see methodology section), to the p*K*a values of carboxylic acids. Physically, the obtained value of *V*_S,max_ on the acidic hydrogen atom reflects the attractive interaction between it and a water molecule, and thus in turn can be used to describe the deprotonation process in electrostatic terms.

## 2. Results

**Figure 1:**
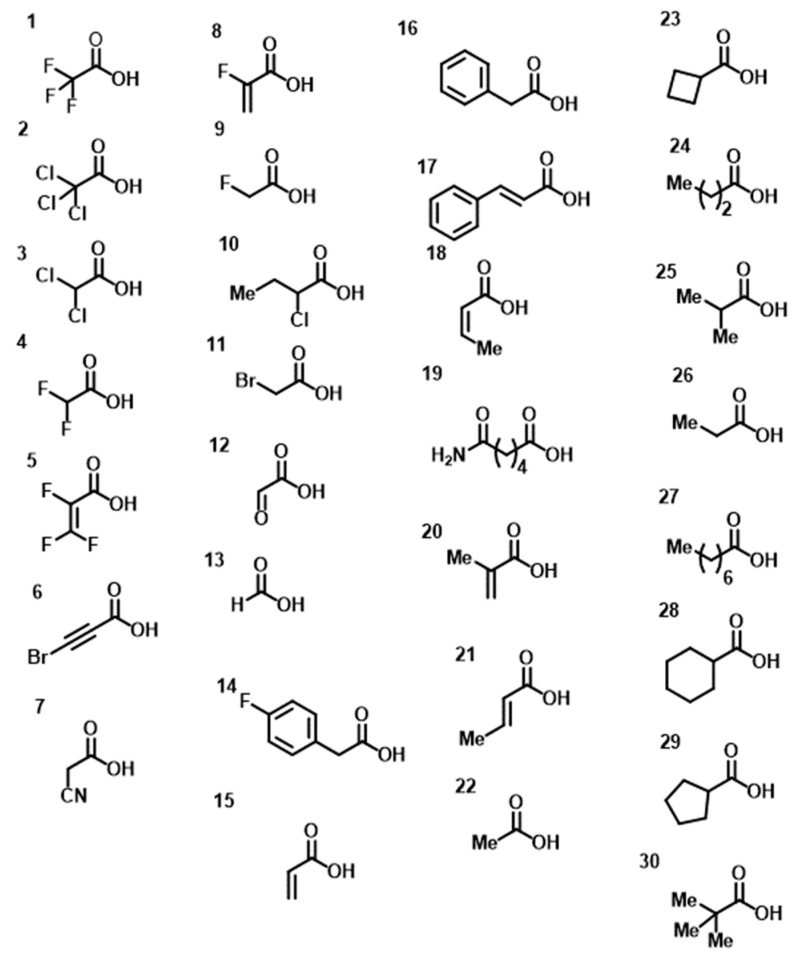
Thirty carboxylic acids comprising the chemical space under study.

Thirty (30) different carboxylic acids with reported p*K*a values were selected from Lange′s Handbook of Chemistry [61], and they were optimized and the surface electrostatic potential calculated (see methods section for full details). The structures of the acids are shown in Figure 1. The levels of theory used were obtained from the combination of the following functionals: ωB97X-D (A) [62], B3LYP (B) [63], LC-ωPBE (C) [64], M06-2X (D) [65] and PBE0 (E) [66], as well as the Møller-Plesset second-order perturbation theory, MP2 (F), and the following basis sets, 6-31+G(*d*,*p*) (1), 6-311++G(*d*,*p*) (2), cc-pVDZ (3), cc-pVTZ (4), aug-cc-pVTZ (5), and Def2-TZVP (6).

**Figure 2:**
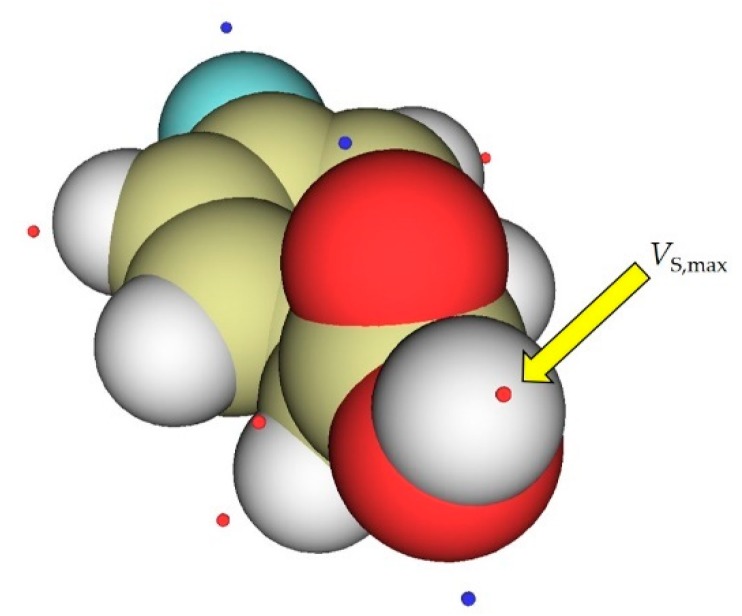
Maximum surface electrostatic potential, *V*_S,max_, over the acidic hydrogen atom shown for compound 14 taking an isodensity value of 0.001 a.u. (isosurface not shown). Red dots represent positive *V*_S,max_ values and the blue dots represent negative *V*_S,max_ values.

In total, thirty-six levels of theory were used to calculate the electronic structure of the thirty carboxylic acids, which comprised the chemical space under study for a total of 1080 different wave functions, upon which the maximum surface potential, *V*_S,max_, was calculated and plotted against the experimental p*K*a_exp_ value. Our model was based on simple linear regressions to obtain the best fittings. The *V*_S,max_ on each acidic hydrogen atom was used for the correlations, as an example, Figure 2 depicts the location of *V*_S,max_ on the acid hydrogen atom for compound 14. This value was calculated on the isodensity surface ρ = 0.001 a.u., and it was used as a descriptor for the magnitude of the attractive interaction RCOOH···H_2_O.

All the correlation coefficients, slopes, and intercepts for all thirty-six levels of theory are collected in Table 1.

**Figure 3:**
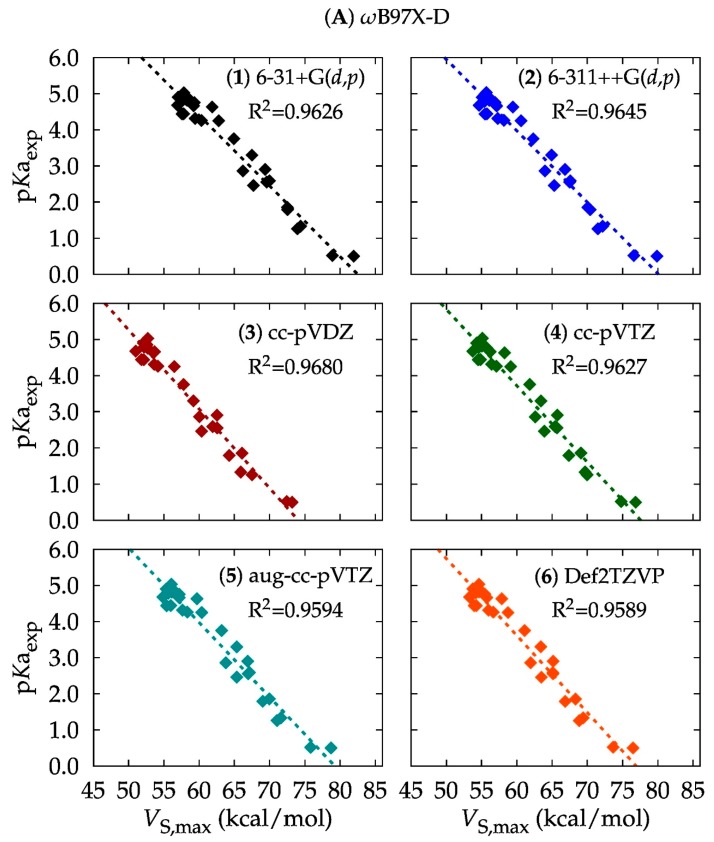
Linear correlations between p*K*a_exp_ against *V*_S,max_ for DFT method (A), with the six basis sets (1) through (6).

The obtained linear model is shown in Figure 3 for method (A) only, the plots with the rest of the methods (B)–(F) are presented in the Supporting Information section (Figures S3, S5, S7, S9 and S11).

A physical interpretation of the trends observed in Figure 3 can be rationalized in terms of the polarization of the O–H bond in the carboxylic acid motif. When the electron density of this bond was more polarized towards the oxygen atom, then the hydrogen atom possessed a more positive electrostatic potential, at the same time it was more labile and readily available for water to abstract it, thus having a lower p*K*a.

**Figure 4:**
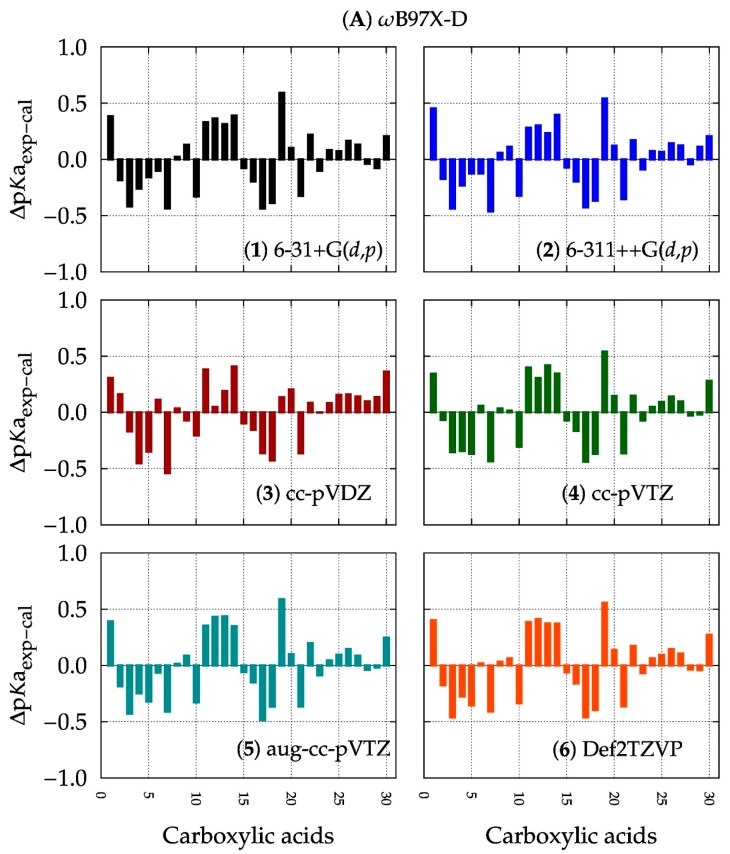
Δp*K*a = p*K*a_exp_ – p*K*a_cal_ for DFT method (**A**) with the six basis sets (1) through (6).

To further analyze the obtained models, a comparison between the experimental and calculated p*K*a values was made by calculating the Δp*K*a = p*K*a_exp_ − p*K*a_cal_. Figure 4 shows these plots for the results obtained with the functional (A), where the corresponding Δp*K*a plots for the other levels of theory are collected in the Supporting Information section (Figures S4, S6, S8, S10 and S12).

The set of models obtained for functionals (C) and (A) had the highest correlation values across the basis sets employed (see the discussion in Section 3 for further results analysis). Particularly, the A3 level of theory (ωB97X-D/cc-pVDZ) exhibited simultaneously, a high correlation (*R*^2^ = 0.9680) and the lowest Δp*K*a values. Table S8 shows the p*K*a intervals for all levels of theory and it can be observed that all (C) models have a Δp*K*a interval above 1.0 units, whereas all (A) models have Δp*K*a intervals below 1.0 unit, which means an accuracy of ±0.5 p*K*a units. Considering these results and the calculation parameters supplied (isodensity and grid values), we proposed the following equation:

p*K*a = −0.2185 *V*_S,max_ + 16.1879 (2)

**Figure 5:**
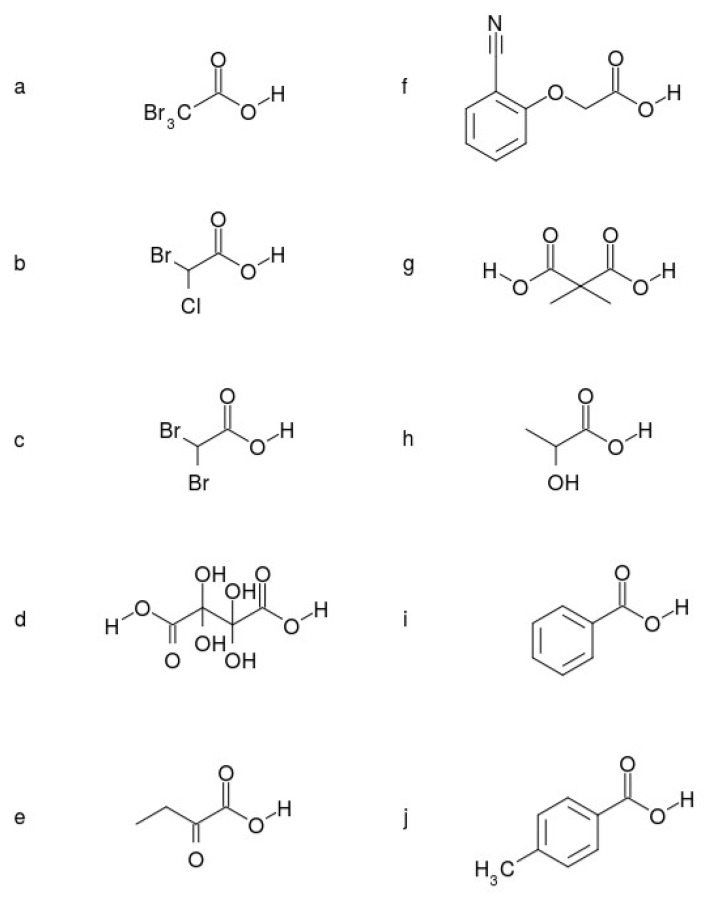
Carboxylic acids used as a test set for Equation (2).

**Figure 6:**
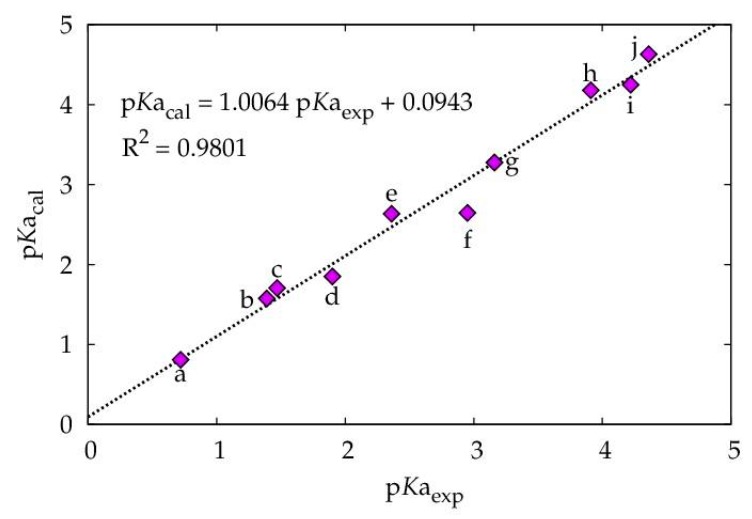
Correlation between the experimental and calculated p*K*a values.

To assess the predictive capabilities of our model, given by Equation (2), we built a test set with ten carboxylic acids (Figure 5), with p*K*a values that lay within the range covered by the original training set. The experimental p*K*a values were reported in Reference [61] and were reproduced in Table 2, together with the calculated values for the test set and the differences, which lay in the range of Δp*K*a = ±0.3 units. Figure 6 shows the remarkable correlation between the experimental and calculated values with a correlation coefficient *R*^2^ = 0.9801.

## 3. Discussion

### 3.1. Computational Method: DFT or Ab Initio?

As a comparison standard, the Møller-Plesset second-order perturbation theory, MP2 (F), was included in the study, not only to assess its accuracy, but to compare the DFT and at least one wave function method as well. From all the tested levels of theory, the highest *R*^2^ correlation coefficients (Table 1) between *V*_S,max_ and p*K*a_exp_ values were obtained consistently with the ab initio MP2 method. Nevertheless, the DFT functional ωB97X-D functional (A), yielded comparably similar results at a fraction of the computational cost. The lowest correlation coefficients were obtained with the B3LYP functional (B), which, despite being one of the most popular ones to model organic molecules, could be describing the surface electrostatic potential inadequately. A similar performance to that of B3LYP was observed for the PBE0 functional (E), which in turn, was slightly improved when long range corrections were included in the case of LC-ωPBE (C). The latter functional was thought to yield much better results due to this long-range correlation term; however, that was not the case.

The M06-2X functional (D) also showed to be properly describing the surface electrostatic potentials, as shown in the high correlation coefficients. This was plausibly because of the dispersion terms included in its formulation. The ωB97X-D functional included an empirical dispersion term which was added a posteriori to correct the energy, but not the electron density [67].

Although the M06-2X functional is widely used and regarded as probably the best functional to model organic reactions [65], in this case, it yielded a larger discrepancy in the Δp*K*a plots than the plots obtained with ωB97X-D (Figure 4 and Figure S8).

The fact that the MP2/cc-pVDZ and the ωB97X-D/cc-pVDZ yielded comparable results showed that for the case of modeling surface electrostatic potentials, a computationally expensive method may not always be preferred, as very similar or even better results can be obtained with a less demanding approach in just a fraction of the time.

### 3.2. Basis Set: Is Larger Better?

Most of the reported benchmarks to model organic molecules deal with the selection of the proper DFT functional methods [68,69,70]. However, little attention is paid to the basis set, or more precisely, to the proper functional/basis set combination (i.e., the level of theory). For further details, refer to Figures S13–S18, where the obtained models are organized by basis set.

Four out of the six methods yielded the strongest *V*_S,max_-p*K*a correlations when using the relatively medium size cc-pVDZ basis set. Surprisingly, the M06-2X functional presented the largest Δp*K*a deviations when combined with the largest basis set aug-cc-pVTZ (Figures S7 and S8).

In the case of the MP2 calculations (Figure S12), increasing the so-called quality of the basis set may not be beneficial in all cases. When comparing the split-valence Pople’s basis sets, practically the same correlation was found with the double-ζ set and the corresponding triple-ζ quality one, 0.9613 versus 0.9625, respectively. On the other hand, the Dunning–Huzinaga basis showed a decrease in correlation when increasing the set size from cc-pVDZ to cc-pVTZ, 0.9702 and 0.9661, respectively. However, the Δp*K*a deviations were practically consistent among the MP2 levels of theory.

In terms of the difference between the experimental and correlated p*K*a values, the A3 level of theory yielded the smallest Δp*K*a deviations, with most of the differences kept under 0.5 p*K*a units, showing that, for this case, a larger basis set size may not always be better.

### 3.3. A Final Remark

**Figure 7:**
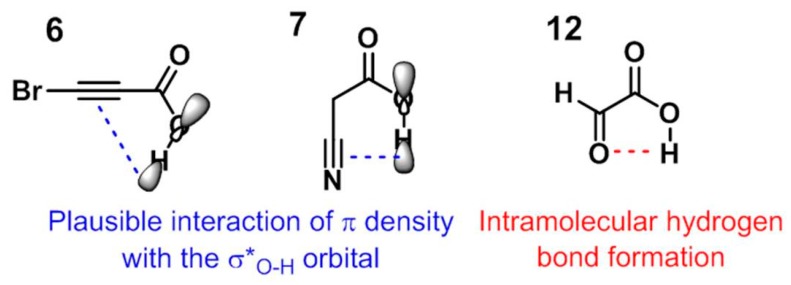
Intramolecular interactions for compounds 6, 7, and 12, conformers at D1 = 180.0°.

In the thirty-six levels of theory tested in this study, the calculation of the *V*_S,max_ of three compounds (6, 7, and 12) required an average of conformers, where the angle (D1 = O=C-O-H) was either 0.0° or 180.0°. The conformation D1 = 180.0° was stable due to strong delocalization effects from nearby п bonds to the σ*_O-H_ orbital in the acidic hydrogen atom or intramolecular hydrogen bonding with Lewis basic motifs (Figure 7). For such kind of compounds, further improvements are required in the methodology for our linear models.

So far, the applicability domain of these regressions is limited by the p*K*a data used to construct the models (0.5 < p*K*a < 5.0). Caution must be taken when using the linear models presented herein for molecules outside this range.

## 4. Materials and Methods

Geometry optimizations and wave function printouts for the 30 carboxylic acids were performed using the Gaussian 09 rev. E01 suite of programs as in Reference [71], at each of the different levels of theory (see text). All calculations included the Conductor-like Polarizable Continuum Model (CPCM) implicit solvation model (water) as described in References [72,73]. The radii for cavity construction was the UFF default which takes the radii from the UFF (Universal Force Field) scaled by 1.1 with explicit spheres for hydrogen atoms. Frequency analyses were performed at the end of each geometry optimization at the same level of theory to verify that the found geometries corresponded to the energy minima. The ultrafine integration grid was used in all the calculations.

The maximum surface potential (*V*_S,max_) calculations were performed on the wave function files with the ‘MultiWFN’ program, version 3.3.8 as in Reference [74], using an isodensity value of 0.001 a.u. All the computed values were collected in the Supporting Information (Tables S1–S6).

## 5. Conclusions

*V*_S,max_ is a scalar quantity that characterizes a σ-hole, and according to our calculations, it has also proven to be a suitable descriptor to be correlated with the p*K*a value of carboxylic acids, yielding differences in p*K*a of high accuracy. Δp*K*a = ±0.30 when the ωB97X-D/cc-pVDZ level of theory was used to calculate the associated electron density upon which the *V*_S,max_ value was obtained. By means of straightforward DFT calculations with a simple implicit solvation model (CPCM), the value of the *V*_S,max_ could be calculated and Equation 2 obtained herein, could be used to estimate the p*K*a values without the need for a full thermodynamic cycle calculation; thus, avoiding long computations of solvation free energies and other costly quantities which require high accuracy methods.

The ωB97X-D/cc-pVDZ level of theory (A3) yielded the lowest Δp*K*a values, standing as the best choice for estimating the p*K*a of any given acid through the calculation of the *V*_S,max_. Hence, we highly recommend this level of theory for geometry optimization and wave function file print. Care must be taken as the p*K*a value sought after should be between 0.5 and 5.0 pH units, for this is the applicability domain of our resulting equations, given the chemical space covered herein.

Further testing is needed for these regression models to become universal. However, it is important to stress that *V*_S,max_ has turned out to be a powerful descriptor for predicting the p*K*a values of carboxylic acids as it is reflected by low, yet distinguishable differences across all methods studied herein. The presence of intramolecular non-covalent interactions, for example, hydrogen bonding, as well as highly electron-delocalizing groups within the chemical space, are key features to consider in the inclusion of an average of the *V*_S,max_ for the most stable conformers. Our proposed descriptor is also dependent of the isodensity value for the definition of the surface upon which it is calculated, and it is highly recommended to keep the value suggested by Bader et al. [75] of ρ = 0.001 a.u. However, by taking these considerations into account as part of the parametrical requirements of Equation (2), then extremely accurate p*K*a results are obtained in a straightforward fashion.

## Figures and Tables

**Table 1:** Linear regression parameters obtained for the p*K*a vs *V*_S,max_ plots. Intercept units in kcal/mol.

Level of Theory	Slope	Intercept	*R* ^2^	Level of Theory	Slope	Intercept	*R* ^2^
A1	−0.1954	16.1237	0.9626	D1	−0.1987	16.3352	0.9598
A2	−0.1975	15.8213	0.9645	D2	−0.1947	15.5073	0.9598
A3	−0.2185	16.1879	0.9680	D3	−0.2201	16.2212	0.9653
A4	−0.2113	16.3958	0.9627	D4	−0.2082	16.2411	0.9577
A5	−0.2063	16.3542	0.9594	D5	−0.2027	16.0780	0.9535
A6	−0.2131	16.3967	0.9589	D6	−0.2095	15.9993	0.9534
B1	−0.1902	15.1863	0.9494	E1	−0.1953	15.8840	0.9553
B2	−0.1909	14.8019	0.9515	E2	−0.1953	15.3858	0.9511
B3	−0.2191	15.5109	0.9570	E3	−0.2167	15.8799	0.9616
B4	−0.2072	15.4614	0.9521	E4	−0.2118	16.1281	0.9536
B5	−0.1983	15.1505	0.9457	E5	−0.2038	15.8232	0.9490
B6	−0.2030	15.1449	0.9277	E6	−0.2125	15.9739	0.9485
C1	−0.2001	16.4372	0.9654	F1	−0.1996	15.8814	0.9613
C2	−0.1996	15.9700	0.9647	F2	−0.2123	15.9191	0.9625
C3	−0.2272	16.6499	0.9682	F3	−0.2285	16.3778	0.9702
C4	−0.2166	16.7945	0.9633	F4	−0.2198	16.3264	0.9661
C5	−0.2085	16..4776	0.9597	F5	−0.2094	16.0399	0.9550
C6	−0.2162	16.4972	0.9579	F6	−0.2187	16.1635	0.9616

**Table 2:** *V*_S,max_ calculated with the A3 model. Experimental and calculated p*K*a values for compounds **a**–**j** and the differences.

	*V*_S,max_	p*K*a_exp_ *	p*K*a_cal_ **	Δp*K*a_exp − cal_
**a**	70.3733	0.7200	0.8097	−0.0897
**b**	66.8745	1.3900	1.5743	-0.1843
**c**	66.2754	1.4700	1.7052	−0.2352
**d**	65.6112	1.9000	1.8503	0.0497
**e**	62.0236	2.3600	2.6343	−0.2743
**f**	61.9719	2.9500	2.6456	0.3044
**g**	59.0914	3.1600	3.2750	−0.1150
**h**	54.9561	3.9100	4.1787	−0.2687
**i**	54.6370	4.2200	4.2484	0.0284
**j**	52.8925	4.3600	3.6296	−0.2696

* See Reference [61]. ** Values obtained with Equation (2).

## Supplementary Materials

The following are available online, Tables S1–S6: Calculated *V*_S,max_ values for carboxylic H atoms to the different levels of theory studied, Table S7: Reported p*K*a values for carboxylic acids studied. Figures S1, S3, S5, S7, S9 and S11: Correlation of p*K*a_exp_ vs *V*_S,max_. Figures S2, S4, S6, S8, S10 and S12: Difference between the experimental and calculated p*K*a values (∆p*K*a_exp−cal_).
