# Supplementary material for: Calculation of VS,max and Its Use as a Descriptor for the Theoretical Calculation of pKa Values for Carboxylic Acids
Source: Molecules. 2018 Dec 26;24(1):79. doi: 10.3390/molecules24010079 (PMC6337188; doi:10.3390/molecules24010079)
Supplement: Supplementary file 1 [file molecules-24-00079-s001.pdf]

Calculation of $V_{S,max}$ and their use as a descriptor for the theoretical calculation of pKa values for carboxylic acids

Guillermo Caballero-García [1], Gustavo Mondragón-Solórzano [1], Raúl Torres-Cadena [1], Marco Díaz-García [1], Jacinto Sandoval-Lira [1], Joaquín Barroso-Flores [1,*]

[1]Centro Conjunto de Investigación en Química Sustentable UAEM – UNAM, Carretera Toluca–Atlacomulco km 14.5, Unidad San Cayetano, Personal de la UNAM, Toluca 50200, Estado de México, México
*Correspondence: jbarroso@unam.mx; Tel.: +52-722-2766610 Ext 7754

maxporting minormation

Table S 1: Calculated $V_{\mathrm{S,max}}$ values [kcal/mol] for carboxylic H atoms listed by method ωB97X-D (**A**). Basis set complementing the level of theory are listed as follows: 6-31+G(d,p) (**1**), 6-311++G(d,p) (**2**), cc-pVDZ (**3**), cc-pVTZ (**4**), aug-cc-pVTZ (**5**) and Def2TZVP (**6**).

**Acid**	**A1**	**A2**	**A3**	**A4**	**A5**	**A6**
1	81.9446	79.8862	73.1940	76.8540	78.7246	76.4829
2	78.9154	76.6000	72.4325	74.8100	75.8385	73.6744
3	73.9344	71.5178	67.5294	69.9582	71.0618	68.8554
4	74.3960	72.1985	65.9259	69.6775	71.5998	69.4109
5	72.5537	70.4205	64.2838	67.3878	69.0274	66.8744
6	72.5162	70.0896	66.0952	69.0905	69.9622	68.3227
7	67.7055	65.3093	60.3548	63.8961	65.3512	63.4795
8	69.5935	67.4913	62.5745	65.6988	66.9625	65.1372
9	69.9615	67.5895	61.9209	65.4267	67.1425	65.1152
10	66.2080	64.0002	60.0476	62.6203	63.8031	61.9482
11	69.3684	66.8501	62.5421	65.7498	66.9031	65.1311
12	67.4955	64.9370	59.2026	63.4158	65.3574	63.3985
13	64.9331	62.3144	57.7822	61.8249	63.2040	61.0953
14	62.7725	60.6010	56.4967	59.1271	60.3597	58.7408
15	60.3206	58.1793	54.1383	57.1010	58.3318	56.6663
16	59.4465	57.2787	53.6339	56.4070	57.6297	55.9596
17	57.5739	55.4734	52.1160	54.5255	55.3957	53.9464
18	57.8101	55.7694	51.8059	54.8421	55.9663	54.2463
19	61.8596	59.4291	53.5122	58.2457	59.6846	57.8408
20	59.2092	57.1395	53.6776	56.2264	57.1595	55.7275
21	56.9301	54.6533	51.0106	53.7376	54.8346	53.2929
22	59.3176	56.9011	52.7009	55.7867	57.1692	55.4465
23	57.5198	55.4372	52.1730	54.6058	55.6294	54.1572
24	58.3080	56.0980	52.4118	55.0256	56.1269	54.6329
25	58.0658	55.8827	52.5836	55.0628	56.1941	54.6108
26	58.4275	56.1617	52.5026	55.1873	56.3444	54.7625
27	58.1540	55.9566	52.3264	54.8836	55.9478	54.4615
28	57.2426	55.0863	52.0987	54.2776	55.3232	53.7755
29	57.0281	55.8468	52.2551	54.2954	55.3947	53.7246
30	57.8370	55.6849	52.7142	55.1043	56.0663	54.6157

Table S 2: Calculated $V_{S,max}$ values [kcal/mol] for carboxylic H atoms listed by method B3LYP (**B**). Basis set complementing the level of theory are listed as follows: 6-31+G(d,p) (**1**), 6-311++G(d,p) (**2**), cc-pVDZ (**3**), cc-pVTZ (**4**), aug-cc-pVTZ (**5**) and Def2TZVP (**6**).

**Acid**	**B1**	**B2**	**B3**	**B4**	**B5**	**B6**
1	79.2794	77.3060	69.9147	73.8357	75.8656	73.6173
2	75.6824	73.3060	68.9035	71.1954	72.1212	69.8628
3	70.9706	68.5571	64.3208	66.7635	67.7521	65.5033
4	71.7677	69.6030	62.8882	66.6903	68.7285	66.4850
5	69.3326	67.2288	60.5095	64.1303	65.7091	63.7488
6	68.0103	65.8861	61.6889	64.7968	65.3708	66.0007
7	65.0762	62.8076	57.3981	61.2820	62.4420	60.7288
8	66.2185	64.0736	58.9555	62.1446	63.2292	61.5552
9	67.2544	64.8963	58.9021	62.5044	64.1055	62.1661
10	63.1718	60.8773	56.9332	59.3934	60.3017	58.5622
11	66.3336	63.9840	59.4363	62.6447	63.5408	61.9548
12	65.0781	62.5447	56.5181	60.7862	62.4232	62.2701
13	62.4257	60.0547	55.0274	58.9587	60.2161	58.2492
14	59.7498	57.4845	53.4752	55.9016	56.9568	55.4651
15	57.0113	54.8216	50.7748	53.5936	54.5345	53.0220
16	56.4439	54.2493	50.6766	53.2465	54.1814	52.7323
17	52.9083	50.7584	47.5853	49.8551	50.3449	49.1431
18	54.1878	52.1704	48.1527	51.0818	51.8249	50.4003
19	58.5909	56.2695	50.2029	54.8909	56.0404	54.4162
20	55.6534	53.6014	50.2518	52.6288	53.1743	51.9465
21	53.3377	51.0635	47.4016	50.0250	50.7627	49.4087
22	56.4609	54.0875	49.8035	52.7925	53.8732	55.3476
23	54.2132	52.0917	49.1252	51.2397	51.9234	50.6189
24	55.2356	52.9395	49.3777	51.8144	52.6130	51.3101
25	54.9748	52.7754	49.6221	51.8614	52.6066	51.2698
26	55.4780	53.1926	49.5808	52.0611	52.9086	51.5395
27	54.9945	52.7497	49.2329	51.5779	52.3515	51.0883
28	53.9086	51.7863	48.9854	50.8902	51.5660	50.2932
29	53.7046	51.6048	49.1285	50.7303	51.4111	50.1374
30	54.6841	52.5564	49.8108	51.9438	52.4701	51.2389

Table S 3: Calculated $V_{S,max}$ values [kcal/mol] for carboxylic H atoms listed by method LC-ωPBE (**C**). Basis set complementing the level of theory are listed as follows: 6-31+G(d,p) (**1**), 6-311++G(d,p) (**2**), cc-pVDZ (**3**), cc-pVTZ (**4**), aug-cc-pVTZ (**5**) and Def2TZVP (**6**).

**Acid**	**C1**	**C2**	**C3**	**C4**	**C5**	**C6**
1	81.7346	79.9230	72.4849	77.0461	78.8029	76.1443
2	78.4190	76.0607	71.5978	74.3122	75.0123	72.4505
3	73.3841	71.0045	66.6382	69.5940	70.3791	67.8078
4	74.2503	72.2558	65.3982	69.9256	71.6815	69.0064
5	73.0084	70.9680	64.1067	68.2444	69.6204	67.1378
6	73.2243	71.0251	66.4345	70.1858	70.7165	68.4253
7	67.4783	65.1224	59.7371	63.7514	64.7286	62.6051
8	69.9060	67.8008	62.5501	66.3051	67.1883	64.7747
9	69.9458	67.7228	61.5557	65.7931	67.1894	64.7582
10	65.8239	63.6523	59.5867	62.5445	63.2887	61.1151
11	68.8905	66.5443	61.9746	65.6547	66.3799	64.2782
12	67.0101	64.6277	58.2243	63.1179	64.7205	62.7854
13	64.7785	62.5698	57.4546	62.0345	63.0477	60.6501
14	62.7464	60.5615	56.3712	59.4348	60.2537	58.2812
15	60.6139	58.4549	54.2187	57.6858	58.4469	56.3979
16	59.2050	57.2313	53.6498	56.7006	57.4556	55.4873
17	58.5433	56.3132	52.7753	55.7237	56.0841	54.3141
18	58.2592	56.2356	52.0850	55.5317	56.1518	54.1835
19	61.8999	59.5760	53.3380	58.7859	59.7054	57.5512
20	59.5956	57.4877	53.8874	56.8968	57.3005	55.5827
21	57.4727	55.1677	51.3119	54.5438	55.1105	53.2449
22	59.3057	56.9889	52.4982	56.1441	57.0968	55.0238
23	57.8753	55.6719	52.2346	55.1666	55.7920	53.2326
24	58.3780	56.1174	52.2970	55.3973	56.0690	54.2207
25	58.1777	56.0084	52.5284	55.4683	56.0887	54.2420
26	58.4876	56.2911	52.3764	55.5691	56.2588	54.3819
27	58.1859	55.9766	52.2092	55.1904	55.8507	54.0344
28	57.4270	55.2952	52.1360	54.7089	55.2754	53.4808
29	57.3424	55.2321	52.4020	54.8106	55.2013	53.4866
30	57.9099	55.7751	52.6629	55.4495	55.9509	54.1903

Table S 4: Calculated $V_{S,max}$ values [kcal/mol] for carboxylic H atoms listed by method M06-2X (**D**). Basis set complementing the level of theory are listed as follows: 6-31+G(d,p) (**1**), 6-311++G(d,p) (**2**), cc-pVDZ (**3**), cc-pVTZ (**4**), aug-cc-pVTZ (**5**) and Def2TZVP (**6**).

**Acid**	**D1**	**D2**	**D3**	**D4**	**D5**	**D6**
1	82.0137	79.7750	73.1054	77.8987	79.4253	76.2984
2	78.6021	75.7083	71.4247	74.4386	75.1063	72.4557
3	73.5169	70.5898	66.5729	69.6098	70.3771	67.6353
4	73.9998	71.4173	65.4506	70.0524	71.6446	68.6590
5	73.0892	70.4747	65.6384	68.6905	69.9032	66.9998
6	72.4801	69.6119	65.8302	69.3072	69.8782	67.9105
7	66.6161	63.8449	59.2792	63.2819	64.2478	61.7701
8	69.7001	66.9811	62.3944	66.1947	67.0394	64.5066
9	69.5873	66.7734	61.4562	65.7631	66.9605	64.2454
10	65.8188	62.8801	59.3279	62.3187	63.0580	60.6188
11	69.2741	66.3157	62.3023	65.8049	66.5560	64.1260
12	67.5258	64.5931	59.3792	63.9229	65.3010	63.2349
13	64.4545	61.5341	57.0898	61.7080	62.6490	59.8722
14	62.7186	59.7958	56.4202	59.3613	60.2231	57.8080
15	60.3921	57.5013	53.8593	57.1834	57.9716	55.7036
16	59.2440	56.3467	53.3409	56.2810	57.1393	54.7890
17	57.9188	54.7913	51.9756	54.7494	55.2354	53.2009
18	58.5109	55.7133	52.1771	55.3041	56.0182	53.7838
19	61.8599	58.6784	53.1593	58.4301	59.4218	56.9886
20	59.4057	56.4941	53.4633	56.3524	56.9063	54.8112
21	57.2516	54.1747	50.8831	53.9706	54.5961	52.5324
22	59.1740	56.1210	52.2917	55.8584	56.7696	54.3959
23	57.5633	54.5249	51.8885	54.5455	55.2390	53.0763
24	58.3388	55.2377	52.0888	55.1002	55.8002	53.6202
25	58.1055	55.0998	52.2911	55.1086	55.7944	53.6129
26	58.4192	55.3951	52.1692	55.2544	55.9909	53.7752
27	58.1874	55.0718	51.9987	54.9138	55.6109	53.4193
28	57.3978	54.2576	51.8391	54.2905	54.9198	52.7565
29	57.6098	54.5823	52.2699	54.7958	55.3647	53.2261
30	57.9739	54.9029	52.4231	55.1037	55.7198	53.5999

Table S 5: Calculated $V_{S,max}$ values [kcal/mol] for carboxylic H atoms listed by method PBE0 (**E**). Basis set complementing the level of theory are listed as follows: 6-31+G(d,p) (**1**), 6-311++G(d,p) (**2**), cc-pVDZ (**3**), cc-pVTZ (**4**), aug-cc-pVTZ (**5**) and Def2TZVP (**6**).

**Acid**	**E1**	**E2**	**E3**	**E4**	**E5**	**E6**
1	80.7651	78.4911	72.1609	75.3976	77.1835	74.8005
2	77.7373	75.0588	71.4053	73.1804	73.9472	71.6094
3	72.8768	70.1097	66.5993	68.5029	69.3312	67.0166
4	73.3089	70.8808	66.5514	68.3128	70.0899	67.7362
5	70.9566	68.4971	62.8142	65.6590	67.0252	64.8624
6	70.5472	67.5979	64.5697	67.1607	67.6210	65.8997
7	66.2579	63.5890	59.0306	62.1665	63.1481	61.3495
8	68.3095	65.8643	61.4366	64.2034	65.0997	63.2052
9	68.8444	66.2566	61.0368	64.1551	65.5218	63.4575
10	65.0533	62.5166	59.1025	61.1617	61.9353	60.0524
11	68.3624	65.7020	61.7733	64.5876	65.3621	63.5476
12	66.3317	63.6362	58.1228	61.9483	63.5163	61.5605
13	64.0789	61.5456	57.0896	60.7903	61.8342	59.7274
14	61.6938	59.1978	55.8076	57.8238	58.7021	57.0460
15	59.1677	56.7198	53.1418	55.7379	56.4988	54.8423
16	58.4294	56.0095	52.8558	55.2012	56.0066	54.3587
17	55.6350	52.3535	50.4186	52.4091	52.8122	51.3753
18	56.4057	54.0506	50.5140	53.1746	53.7664	52.1543
19	60.7208	58.0702	52.4832	56.9759	57.9484	56.1811
20	58.0023	55.6123	52.6546	54.8144	55.2746	53.8519
21	55.5242	52.9593	49.7313	52.1348	52.7141	51.2244
22	58.2795	55.6608	51.8665	54.6052	55.5374	53.8495
23	56.4159	54.0098	51.4100	53.3251	53.8930	52.4038
24	57.2453	54.7071	51.5357	53.7430	54.4133	52.9577
25	57.0395	54.5852	51.7998	53.8075	54.4698	52.9347
26	57.3835	54.9086	51.6840	53.9724	54.6691	53.1282
27	57.0496	54.5496	51.4106	53.5335	54.1960	52.7454
28	56.0998	53.6754	51.2319	52.9149	53.4835	52.0009
29	55.9068	53.5649	51.4603	52.9264	53.3718	51.9372
30	56.7980	54.3904	51.9361	53.8565	54.3562	52.9118

Table S 6: Calculated $V_{S,max}$ values [kcal/mol] for carboxylic H atoms listed by method MP2 (**F**). Basis set complementing the level of theory are listed as follows: 6-31+G(d,p) (**1**), 6-311++G(d,p) (**2**), cc-pVDZ (**3**), cc-pVTZ (**4**), aug-cc-pVTZ (**5**) and Def2TZVP (**6**).

**Acid**	**F1**	**F2**	**F3**	**F4**	**F5**	**F6**
1	79.6544	75.4853	71.1429	74.1600	76.6389	73.9829
2	74.6433	70.6600	69.4179	70.9317	72.4270	69.9180
3	70.1096	66.2176	64.9022	66.5645	67.9951	65.5595
4	72.0066	68.1073	64.0718	67.0248	69.4509	66.8954
5	70.8934	66.8040	62.8101	65.2816	67.3293	64.9780
6	69.8541	64.6769	65.0085	66.0062	67.2257	65.4800
7	64.7651	60.9210	58.4068	60.3333	61.9112	60.2700
8	67.4230	63.9535	61.1404	63.4210	64.9359	62.9059
9	67.7381	63.7904	60.2153	63.0068	65.0517	62.7828
10	63.1749	59.8321	57.8896	59.6048	60.9442	58.9892
11	66.5882	62.4169	60.3988	62.7509	64.1890	62.2684
12	64.8541	60.8955	57.0388	60.1655	62.5186	60.5213
13	62.5403	58.7491	55.9363	59.3208	61.0050	58.7195
14	60.4276	57.0294	54.7923	56.6422	58.2258	56.3779
15	57.8931	54.7821	52.6483	54.7600	56.0504	54.2493
16	56.6893	53.5109	51.7590	53.7738	55.0709	53.2787
17	55.8686	52.9535	51.1211	52.6260	53.5683	52.0652
18	56.2481	53.2734	50.9604	53.0396	54.0867	52.4266
19	57.1655	53.7694	51.6981	53.5171	57.2063	53.2788
20	56.5595	53.6342	52.0621	53.7761	54.7298	53.1519
21	54.9563	51.8968	50.0482	51.8488	52.9278	51.3254
22	56.9155	53.5078	51.1970	53.4146	54.9179	53.0499
23	55.1246	51.9828	50.6525	52.1076	53.2316	51.6334
24	55.8152	52.6118	50.8466	52.5730	53.7826	52.1647
25	55.5544	52.3779	51.0279	52.5903	53.7370	52.0707
26	55.9647	52.7538	50.9891	52.7969	54.0444	52.3415
27	55.5589	52.4499	50.7082	52.3802	53.5396	51.9472
28	54.6498	51.5940	50.4773	51.7087	52.7907	51.1881
29	54.9044	52.0093	51.0602	52.3683	53.3095	51.7899
30	55.2497	52.0371	51.2099	52.5814	53.5240	51.9329

Table S 7: Reported p*K*a values for carboxylic acids studied (see reference [73] of the manuscript).

**Acid**	p*K*a	**Acid**	p*K*a	**Acid**	p*K*a
1	0.5000	11	2.9020	21	4.6760
2	0.5200	12	3.3000	22	4.7560
3	1.2600	13	3.7510	23	4.7850
4	1.3300	14	4.2500	24	4.8170
5	1.7900	15	4.2600	25	4.8530
6	1.8550	16	4.3120	26	4.8740
7	2.4600	17	4.4380	27	4.8950
8	2.5500	18	4.4400	28	4.9000
9	2.5860	19	4.6290	29	4.9050
10	2.8600	20	4.6600	30	5.0310

Table S 8: Maximum and minimum values of the $\Delta pKa_{\text{exp-cal}}$ and intervals for all levels of theory.

	$\Delta pKa_{\text{exp-cal}}$	**1**	**2**	**3**	**4**	**5**	**6**
	min	-0.4369	-0.4631	-0.5390	-0.4371	-0.4859	-0.4637
**A**	max	0.5915	0.5446	0.4080	0.5400	0.5901	0.5581
	int	1.0283	1.0077	**0.9469**	0.9770	1.0760	1.0218
	min	-0.6860	-0.6767	-0.6457	-0.6919	-0.7269	-0.7293
**B**	max	0.5858	0.5661	0.4570	0.5427	0.5938	0.8485
	int	1.2717	1.2428	1.1026	1.2346	1.3207	1.5777
	min	-0.4960	-0.5387	-0.6163	-0.5280	-0.5424	-0.5747
**C**	max	0.5755	0.5494	0.4089	0.5656	0.6009	0.5765
	int	1.0715	1.0881	1.0252	1.0936	1.1433	1.1512
	min	-0.6363	-0.6173	-0.7109	-0.6058	-0.5941	-0.5970
**D**	max	0.5875	0.5459	0.4497	0.5530	0.5966	0.5702
	int	1.2238	1.1631	1.1606	1.1588	1.1907	1.1672
	min	-0.5799	-0.7230	-0.6254	-0.5875	-0.6235	-0.6174
**E**	max	0.6044	0.5844	0.4660	0.5709	0.6141	0.5949
	int	1.1843	1.3075	1.0914	1.1584	1.2376	1.2123
	min	-0.6245	-0.5999	-0.5741	-0.6066	-0.6161	-0.5677
**F**	max	0.5210	0.6078	0.3901	0.4722	0.5677	0.5143
	int	1.1456	1.2077	0.9642	1.0788	1.1838	1.0819

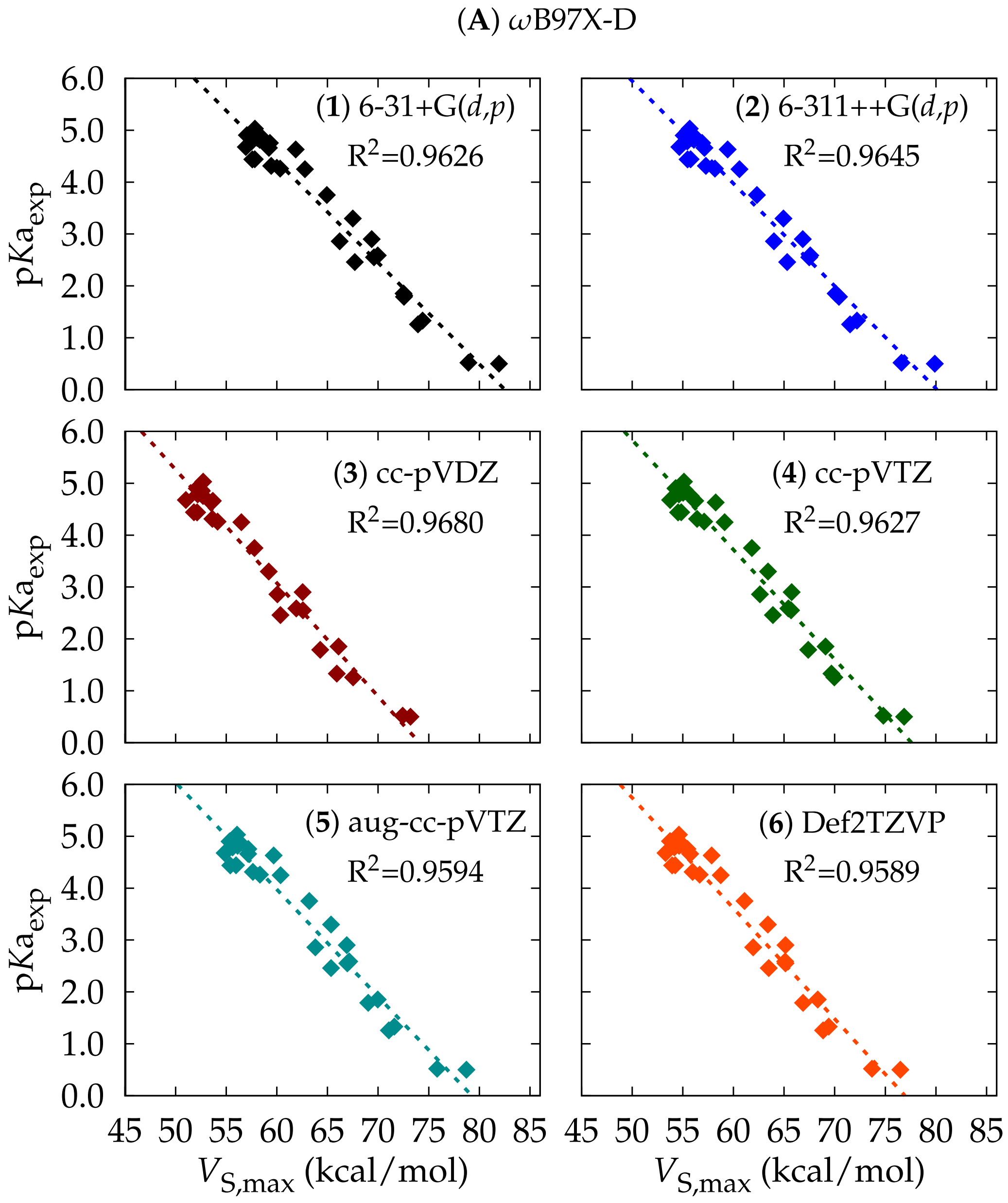

Figure S 1: Correlation of pKa_{exp} vs $V_{S,max}$ with ωB97X-D (**A**)/6-31+G(d,p) (**1**), 6-311++G(d,p) (**2**), cc-pVDZ (**3**), cc-pVTZ (**4**), aug-cc-pVTZ (**5**) and Def2TZVP (**6**). R^2 shown in each panel.

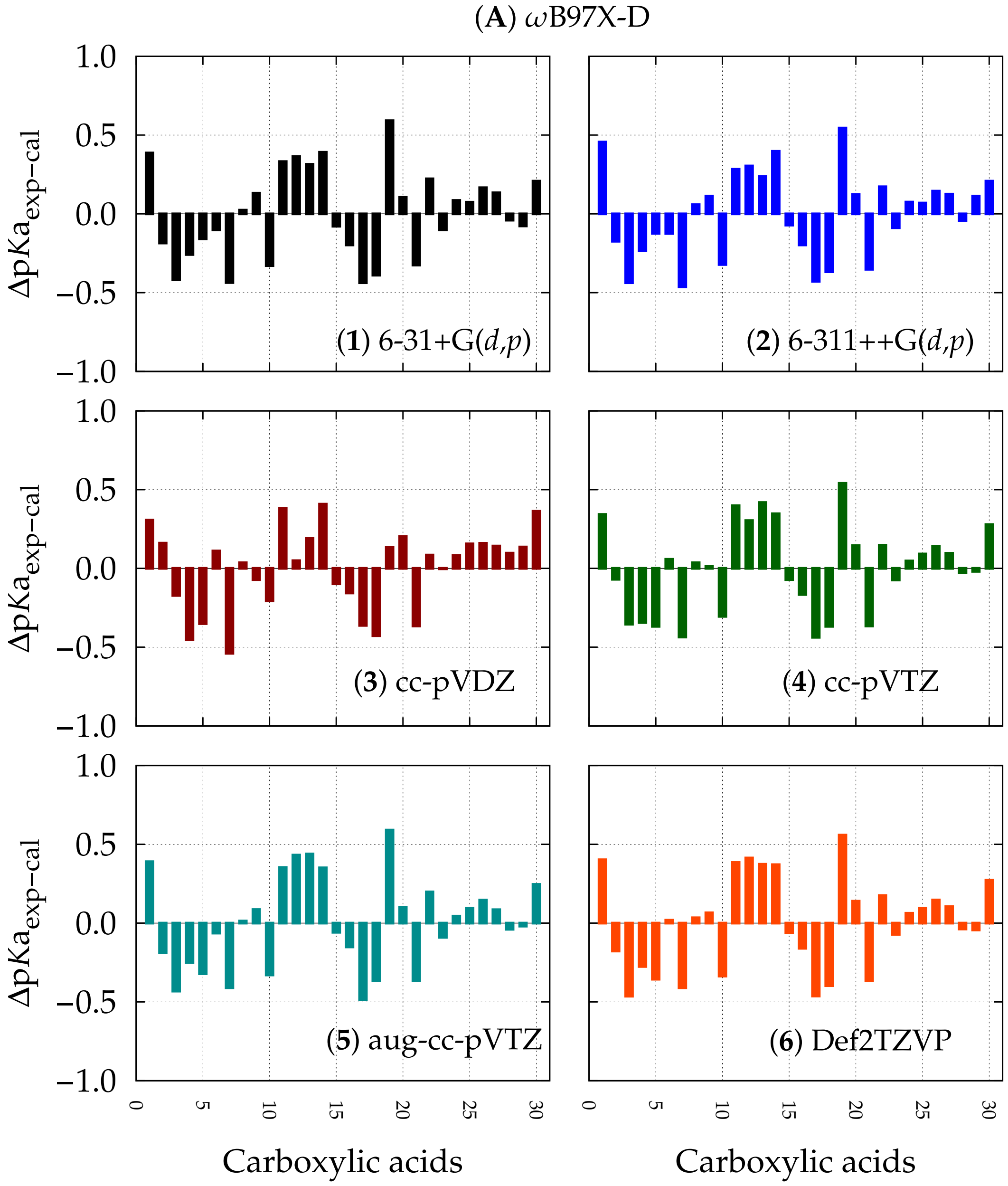

Figure S 2: Difference between the experimental and calculated pKa values ($\Delta pKa_{\text{exp-cal}}$).

(**B**) B3LYP

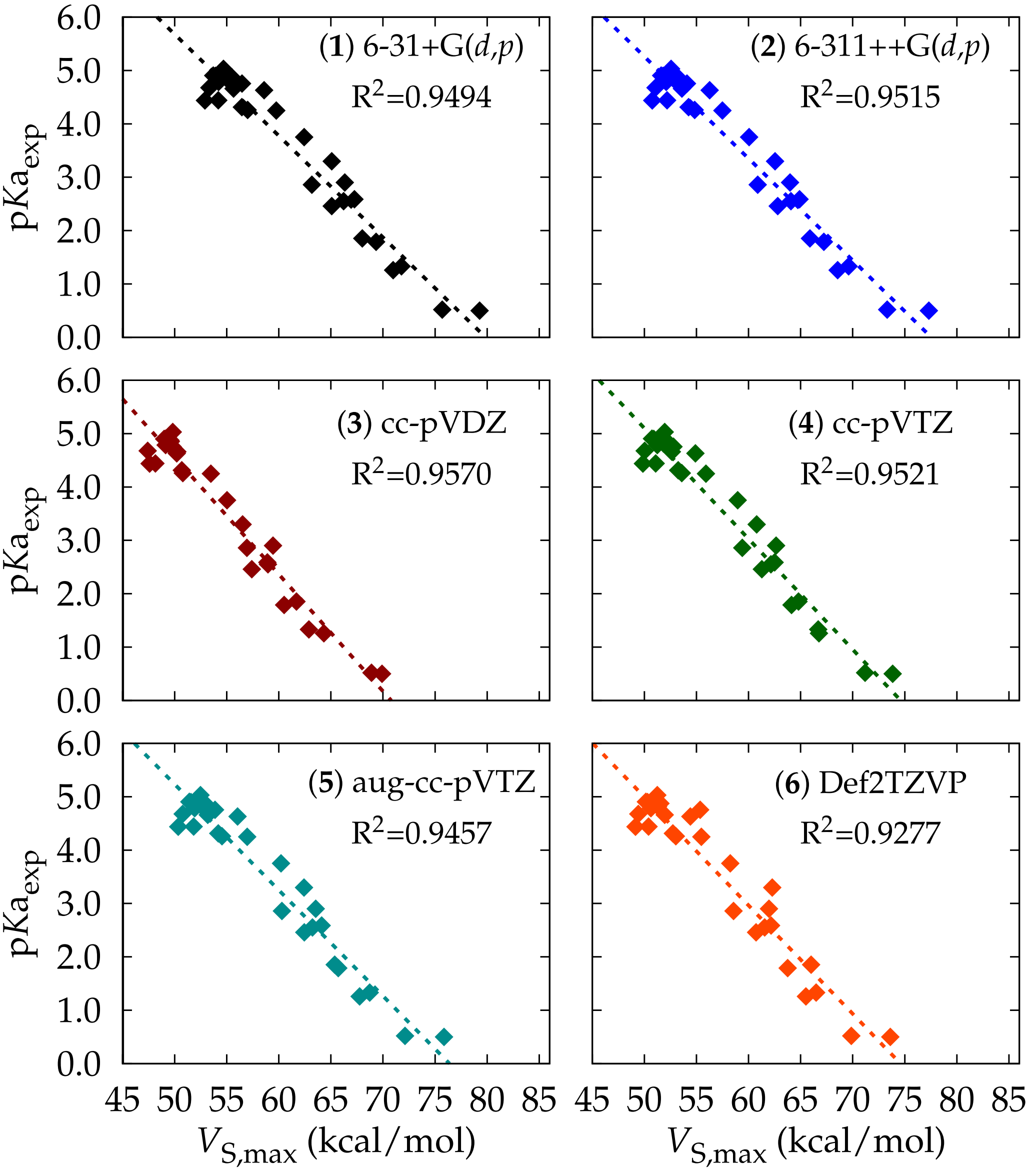

Figure S 3: Correlation of pKa_{exp} vs $V_{S,max}$ with B3LYP (**B**)/6-31+G(d,p) (**1**), 6-311++G(d,p) (**2**), cc-pVDZ (**3**), cc-pVTZ (**4**), aug-cc-pVTZ (**5**) and Def2TZVP (**6**). R^2 shown in each panel.

(**B**) B3LYP

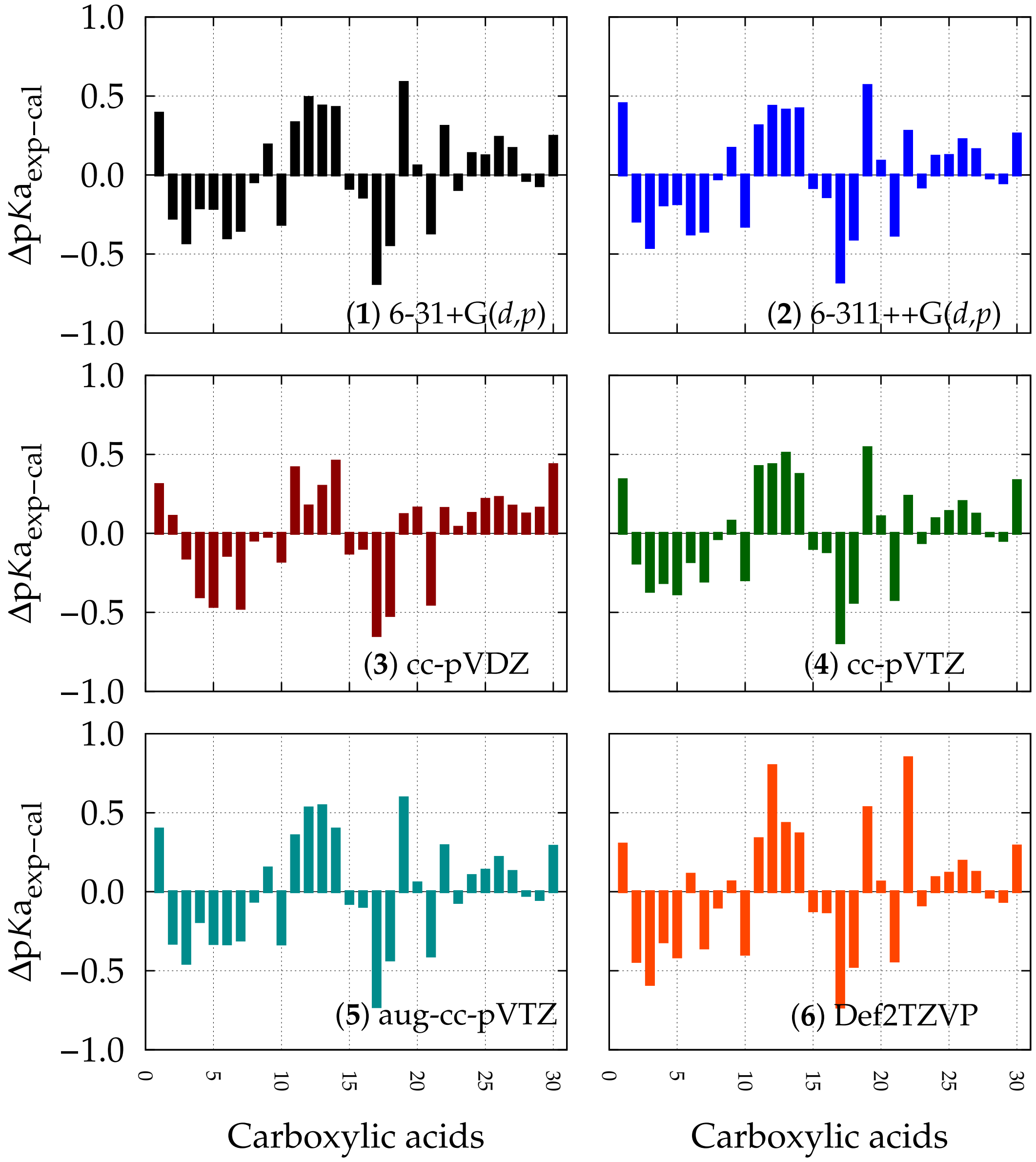

Figure S 4: Difference between the experimental and calculated pKa values ($\Delta pKa_{\text{exp-cal}}$).

(**C**) LC-ωPBE

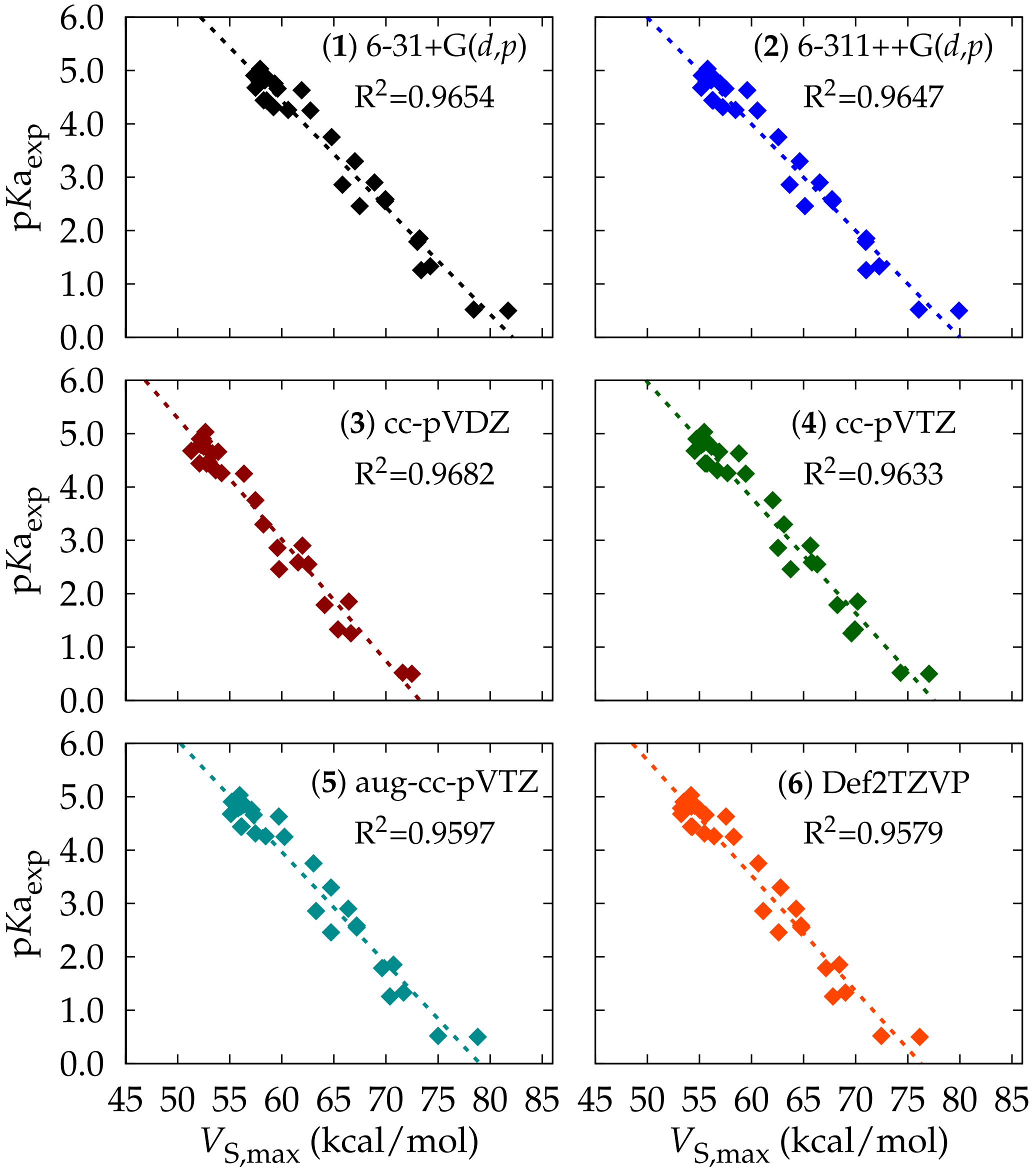

Figure S 5: Correlation of pKa_{exp} vs $V_{\mathrm{S,max}}$ with LC-ωPBE (**C**)/6-31+G(d,p) (**1**), 6-311++G(d,p) (**2**), cc-pVDZ (**3**), cc-pVTZ (**4**), aug-cc-pVTZ (**5**) and Def2TZVP (**6**). R^2 shown in each panel.

(**C**) LC-ωPBE

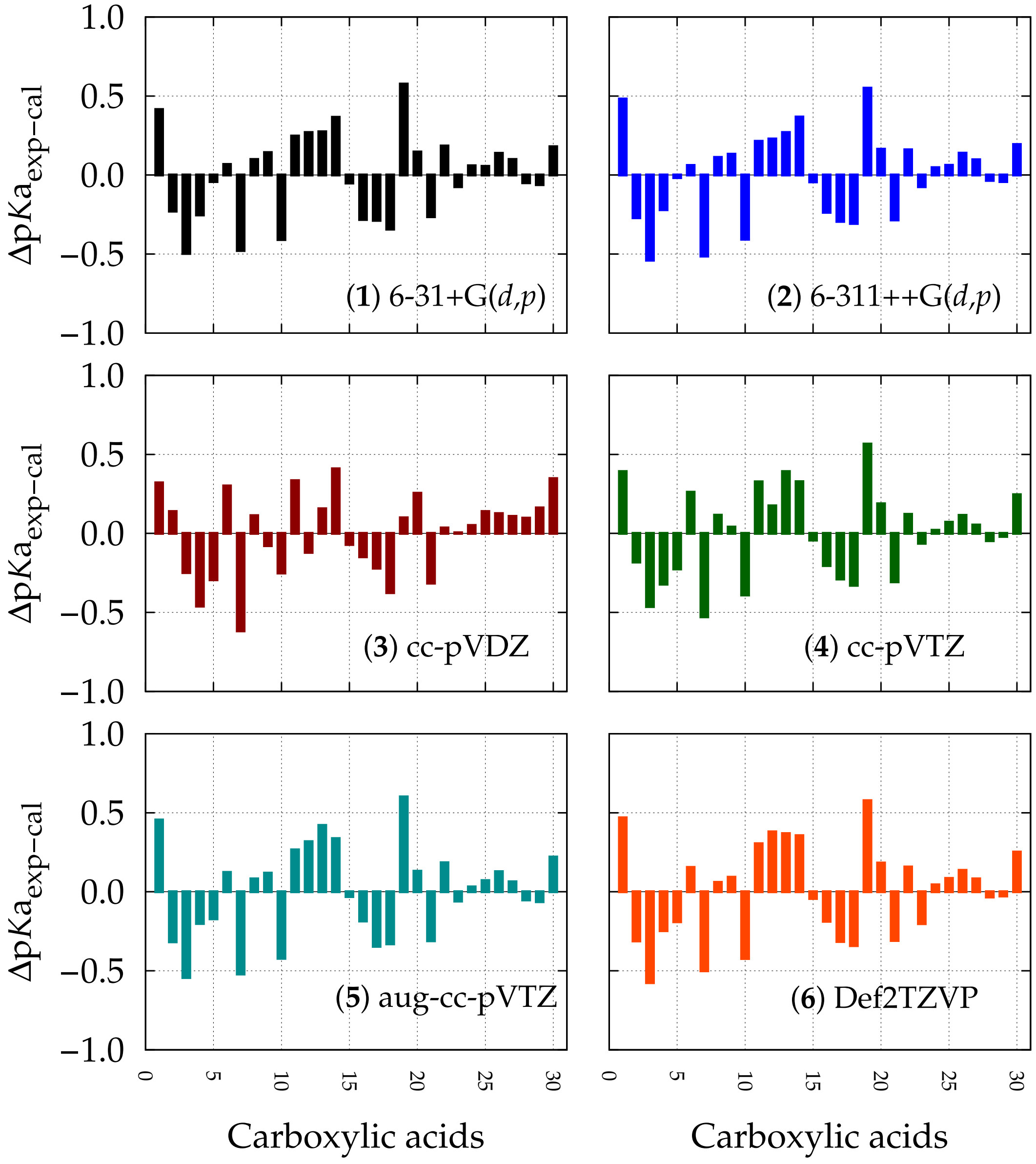

Figure S 6: Difference between the experimental and calculated pKa values ($\Delta pKa_{\text{exp-cal}}$).

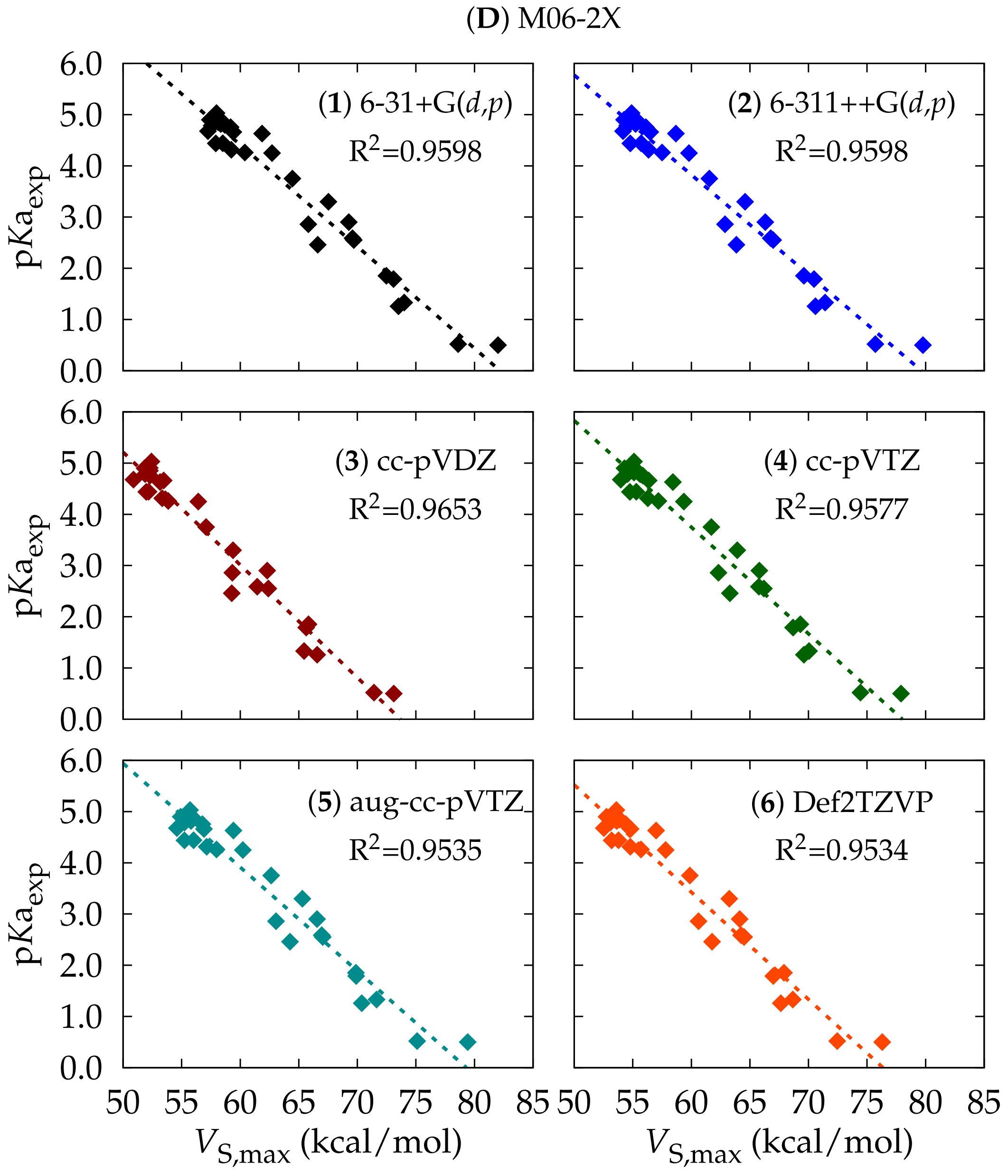

Figure S 7: Correlation of pKa_{exp} vs $V_{S,max}$ with M06-2X (**D**)/6-31+G(d,p) (**1**), 6-311++G(d,p) (**2**), cc-pVDZ (**3**), cc-pVTZ (**4**), aug-cc-pVTZ (**5**) and Def2TZVP (**6**). R^2 shown in each panel.

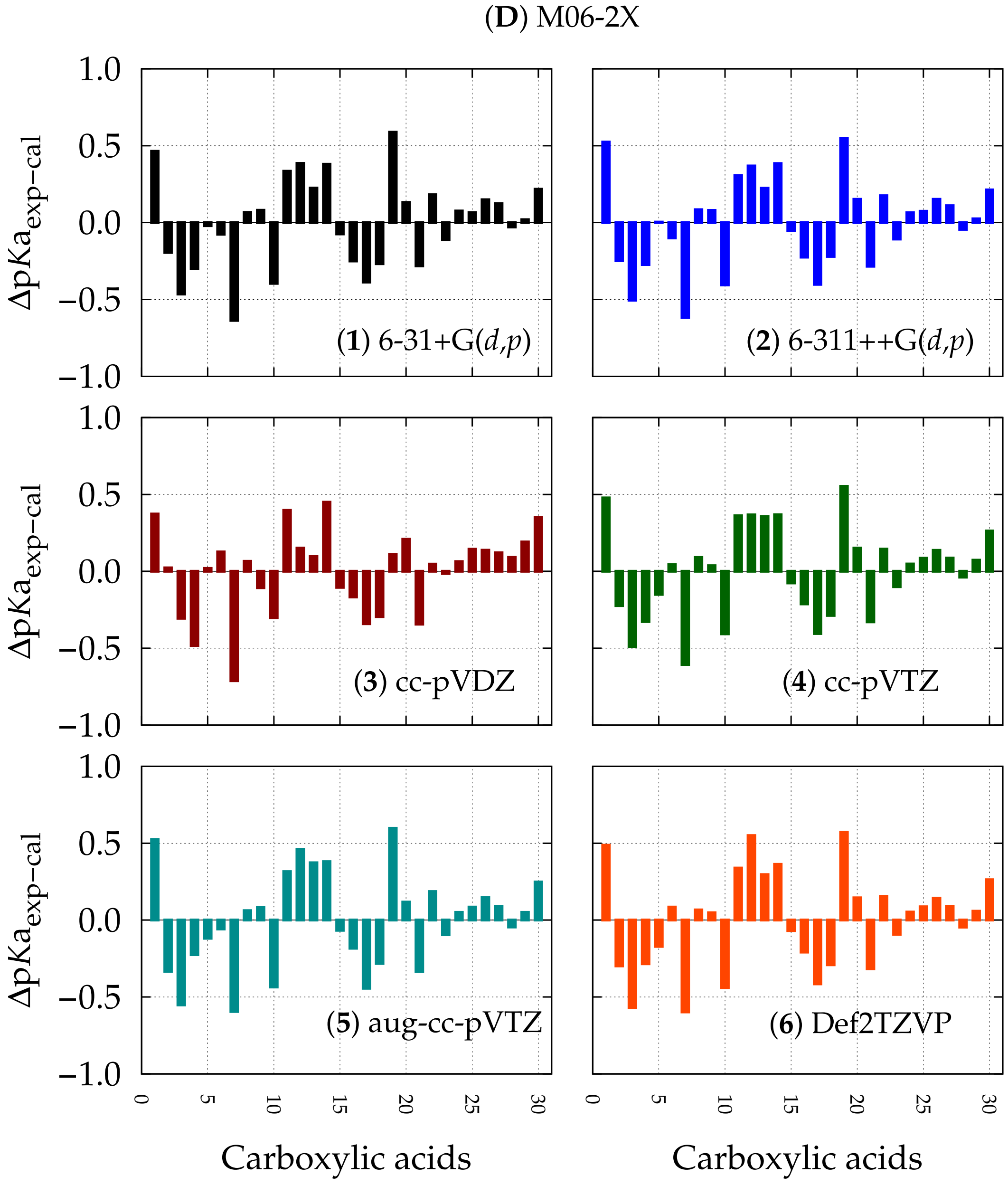

Figure S 8: Difference between the experimental and calculated pKa values ($\Delta pKa_{\text{exp-cal}}$).

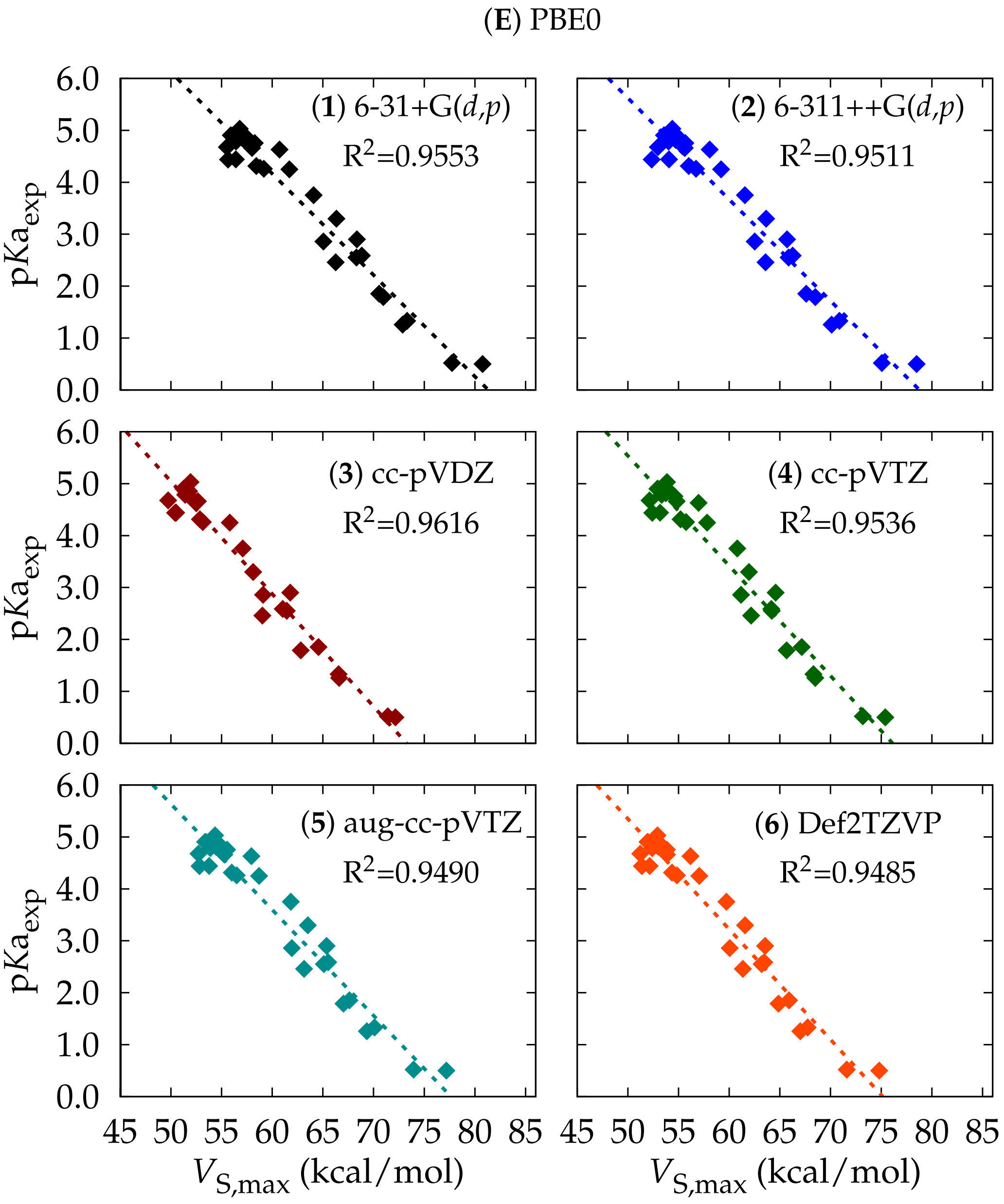

Figure S 9: Correlation of pKa_{exp} vs $V_{S,max}$ with PBE0 (**E**)/6-31+G(d,p) (**1**), 6-311++G(d,p) (**2**), cc-pVDZ (**3**), cc-pVTZ (**4**), aug-cc-pVTZ (**5**) and Def2TZVP (**6**). R^2 shown in each panel.

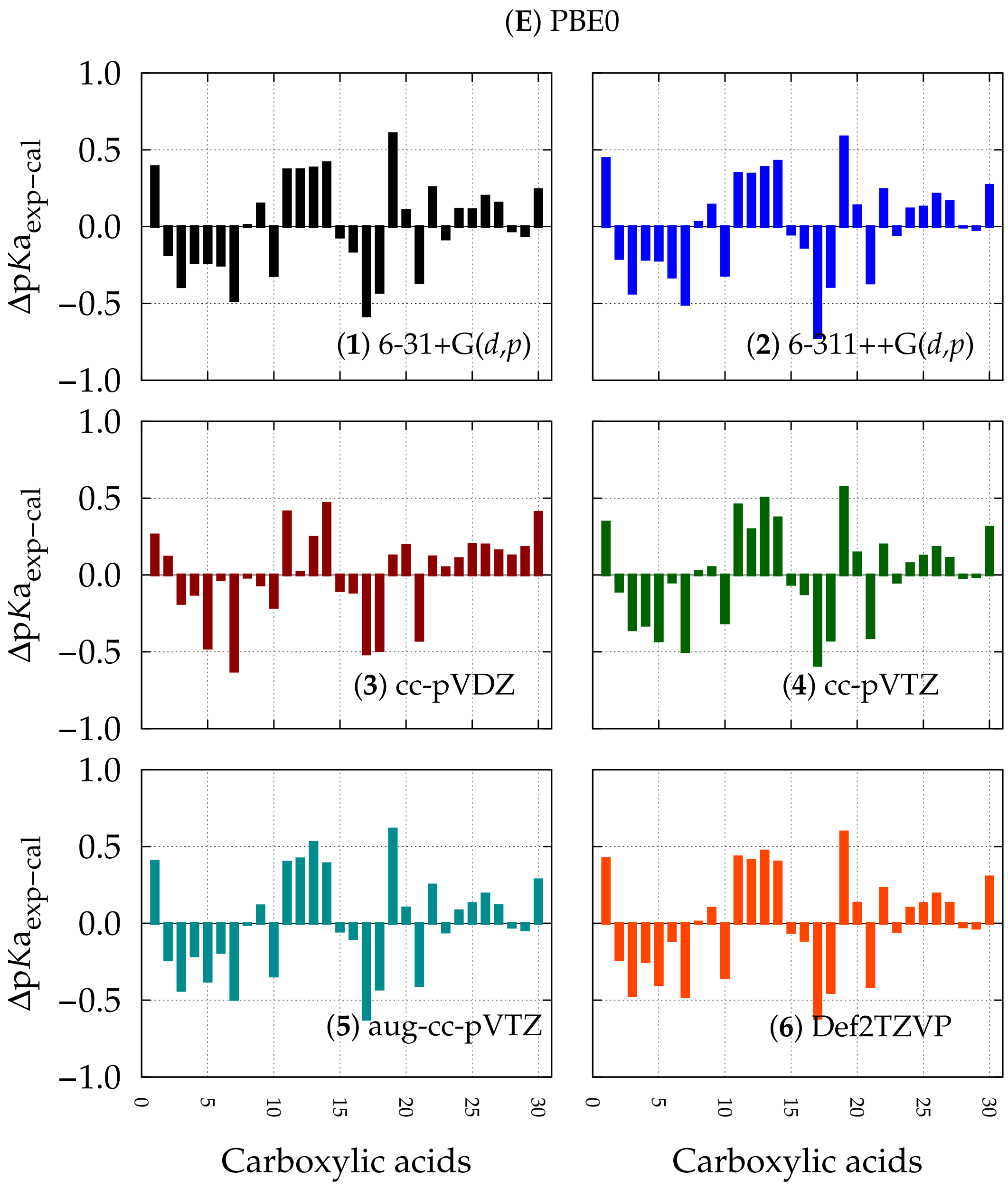

Figure S 10: Difference between the experimental and calculated pKa values ($\Delta pKa_{\text{exp-cal}}$).

(**F**) MP2

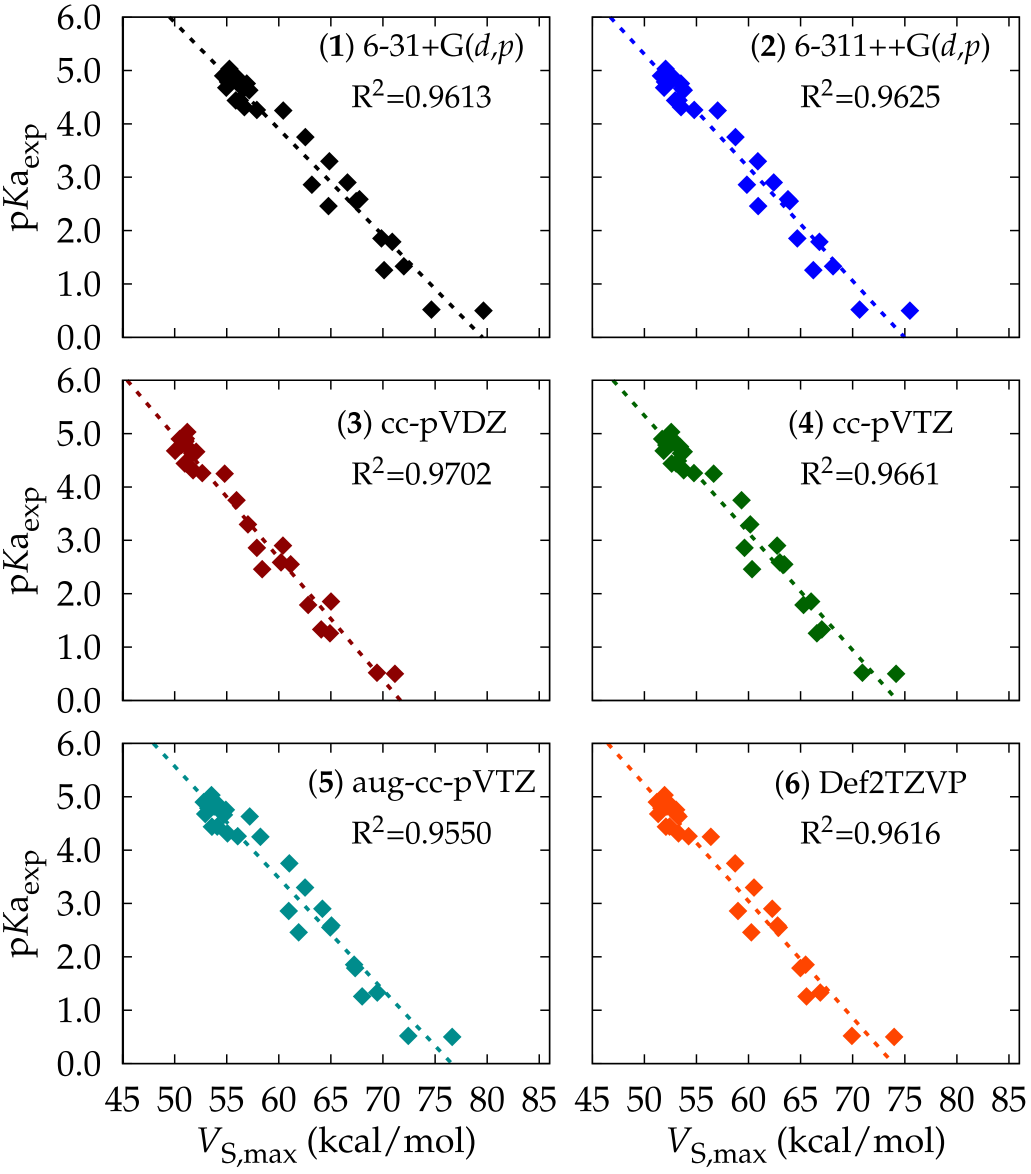

Figure S 11: Correlation of pKa_{exp} vs $V_{\mathrm{S,max}}$ with MP2 (**F**)/6-31+G(d,p) (**1**), 6-311++G(d,p) (**2**), cc-pVDZ (**3**), cc-pVTZ (**4**), aug-cc-pVTZ (**5**) and Def2TZVP (**6**). R^2 shown in each panel.

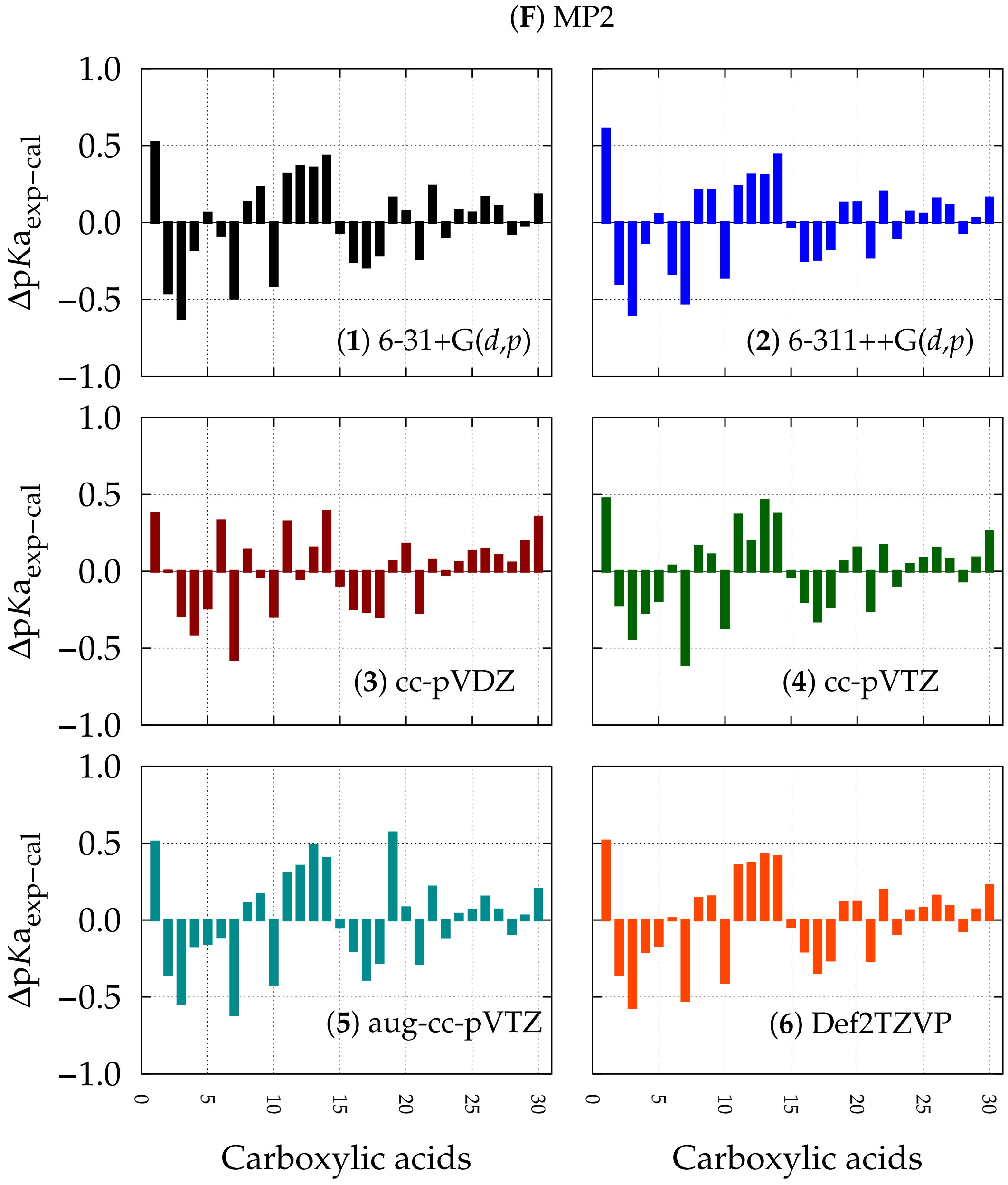

Figure S 12: Difference between the experimental and calculated pKa values ($\Delta pKa_{exp\text{-}cal}$).

(**1**) 6-31+G(*d*,*p*)

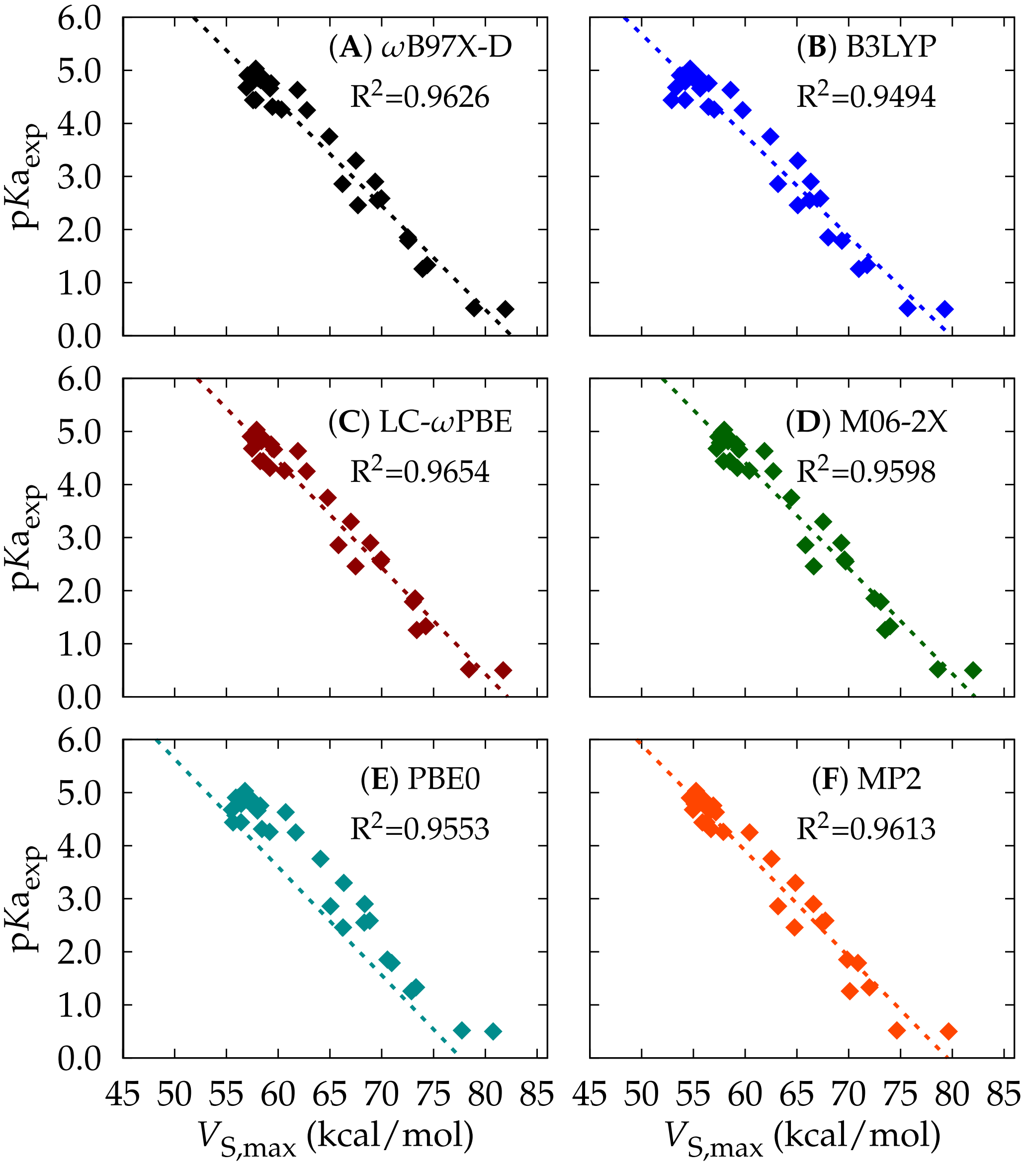

Figure S 13: Correlation of pKa_{exp} vs $V_{\text{S,max}}$. R^2 shown in each panel.

(**2**) 6-311++G(*d*,*p*)

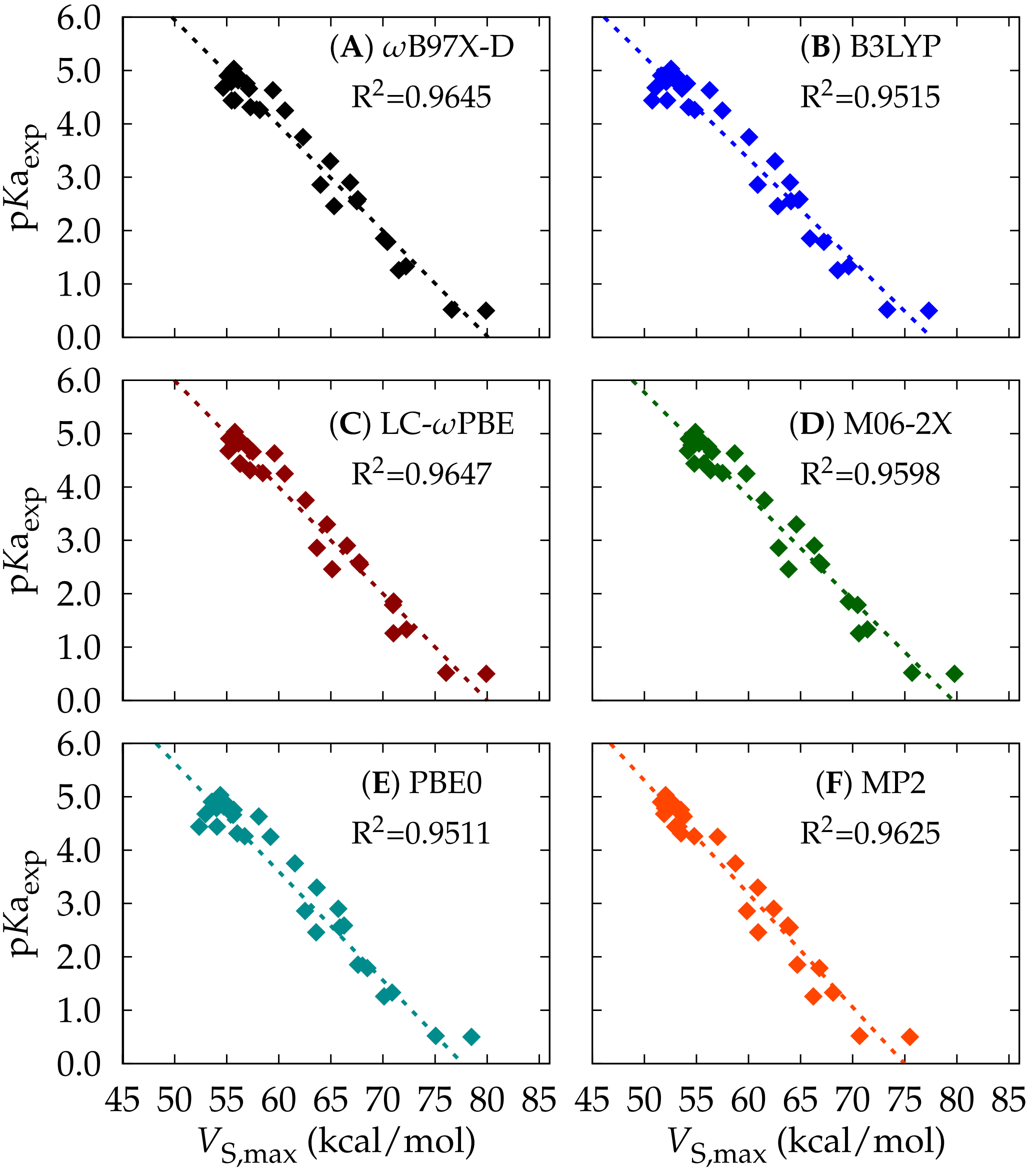

Figure S 14: Correlation of pKa_{exp} vs $V_{S,max}$ with six different methods *vesus* (**2**) 6-311++G(d,p). R^2 shown in each panel.

(**3**) cc-pVDZ

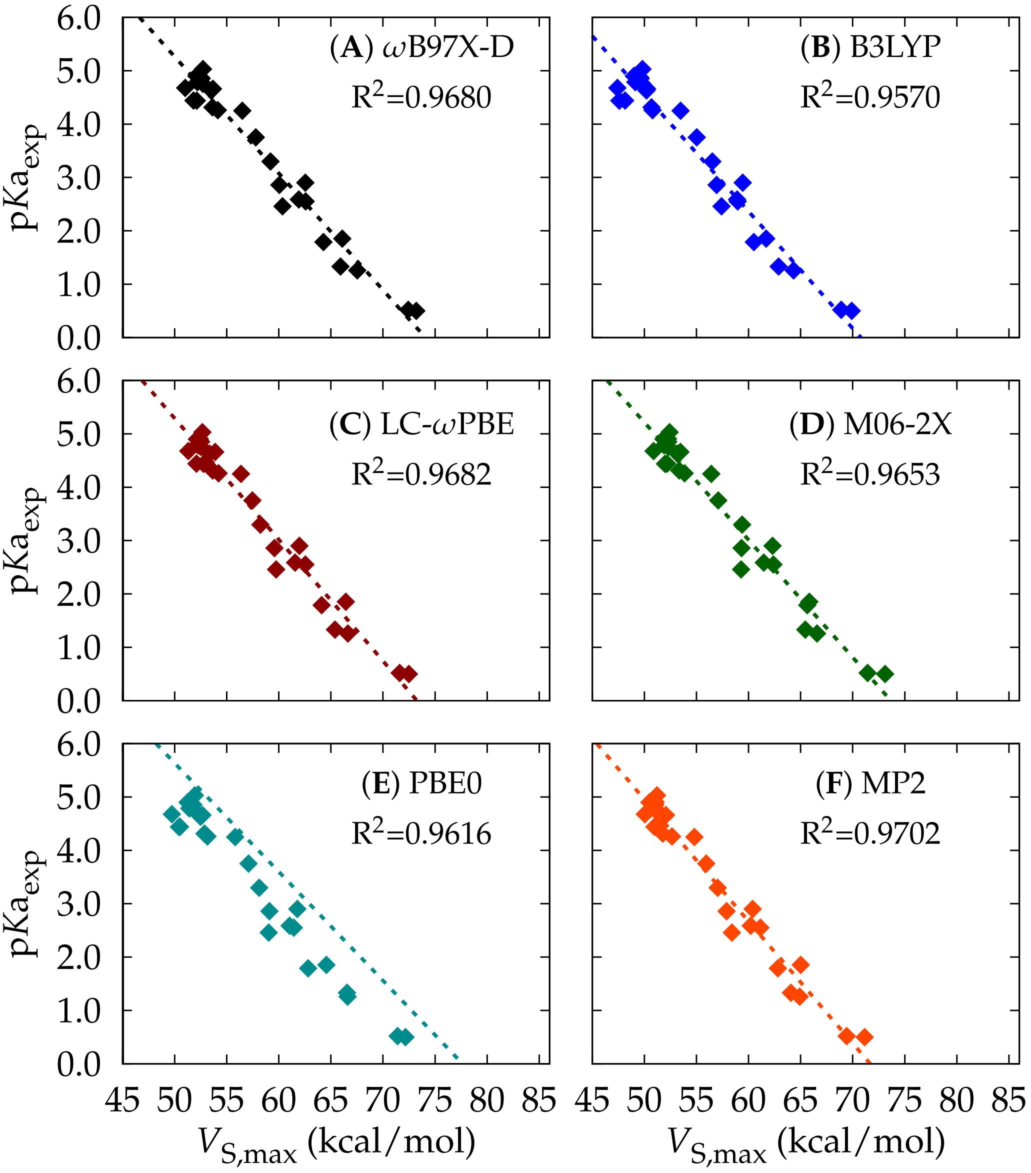

Figure S 15: Correlation of pKa_{exp} vs $V_{S,max}$. R^2 shown in each panel.

(**4**) cc-pVTZ

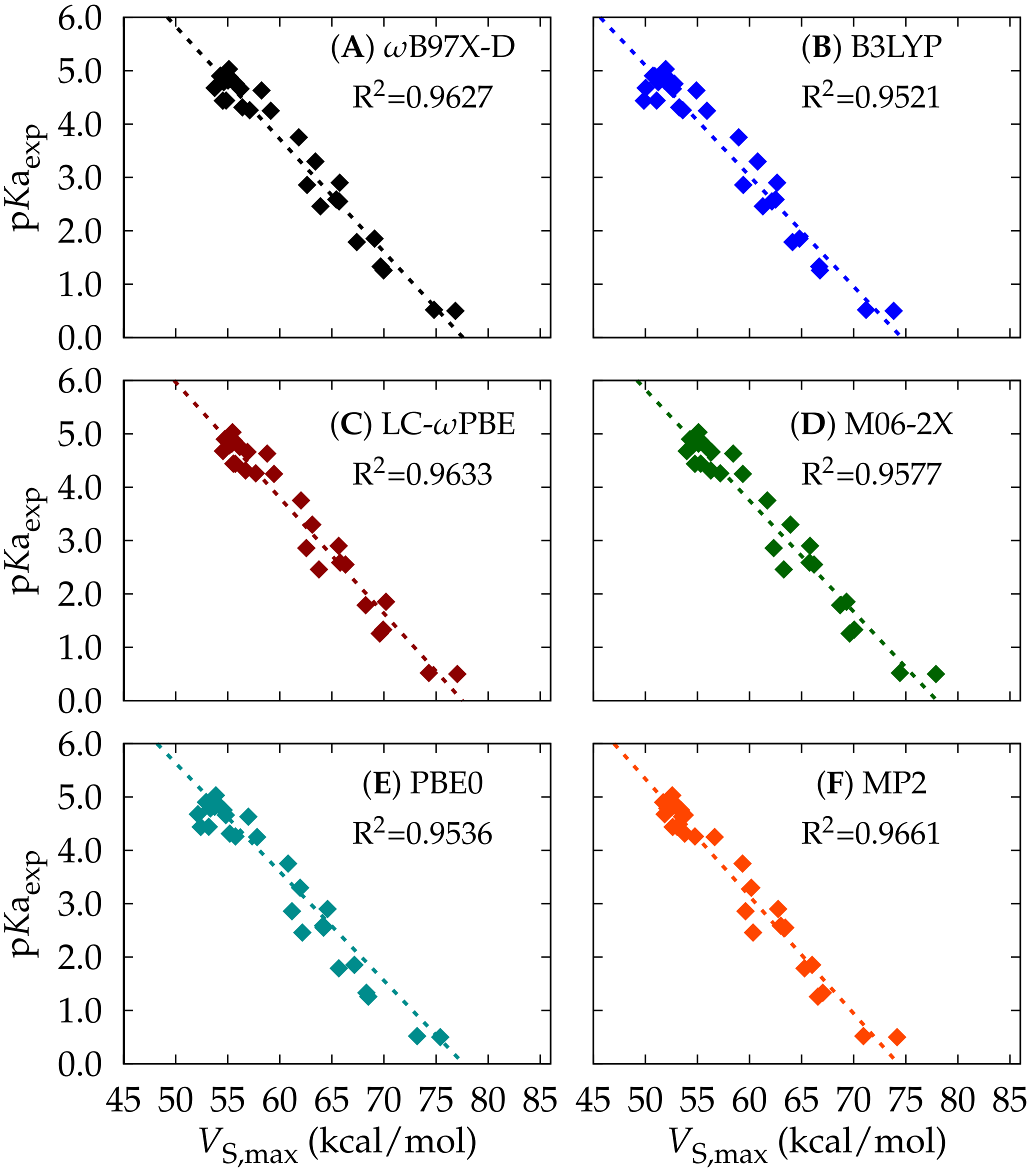

Figure S 16: Correlation of pKa_{exp} vs $V_{S,max}$. R^2 shown in each panel.

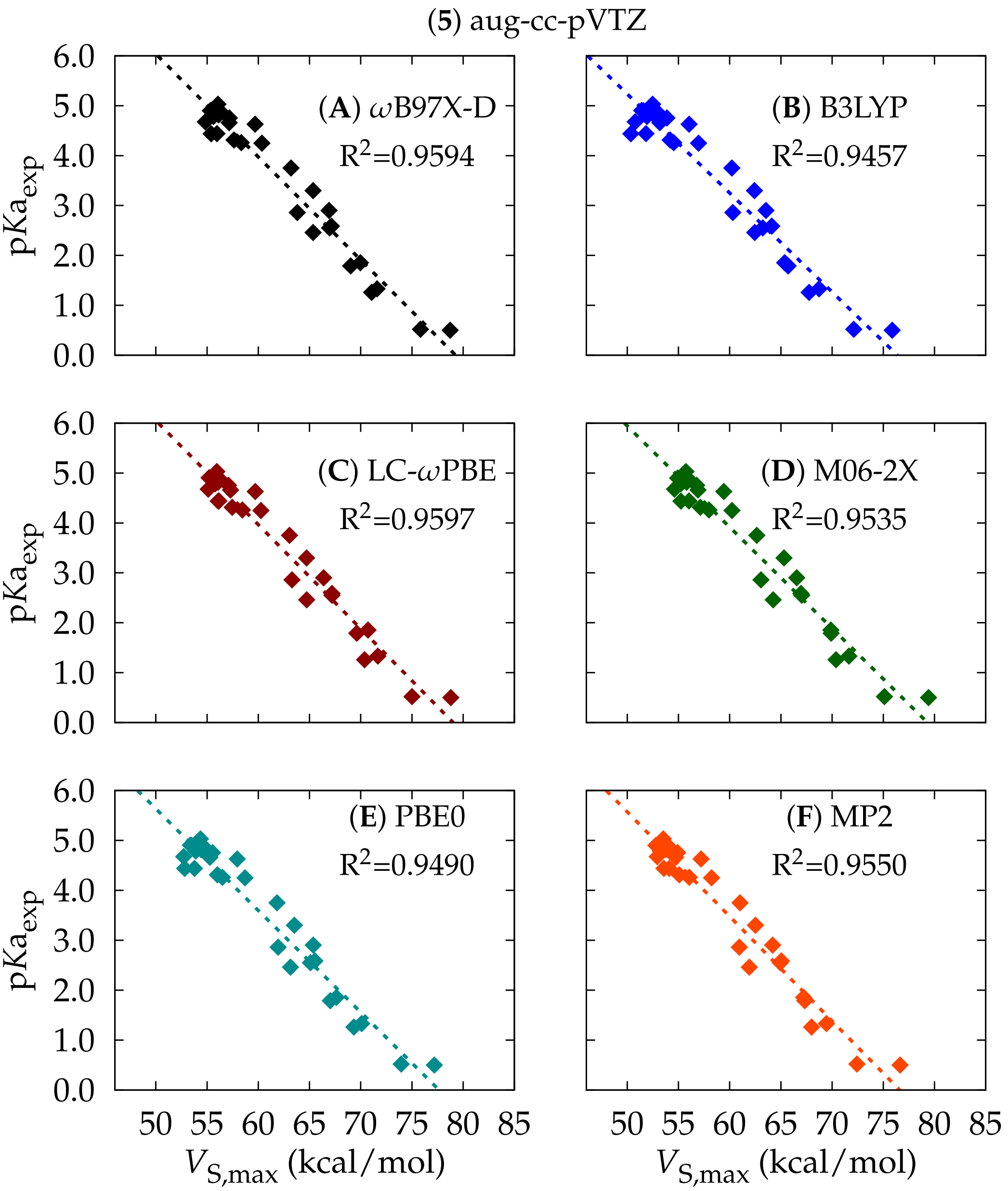

Figure S 17: Correlation of pKa_{exp} vs $V_{S,max}$. R^2 shown in each panel.

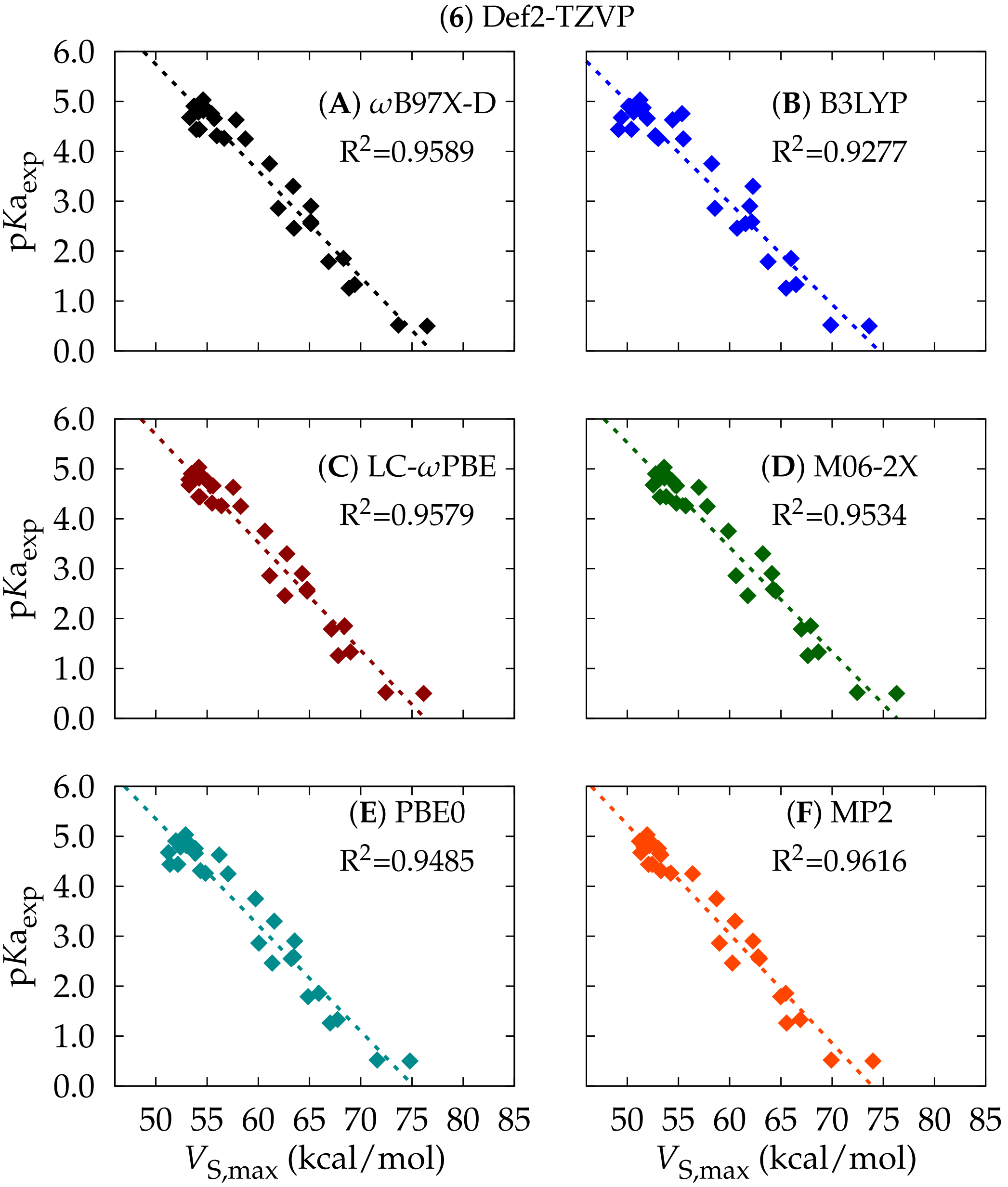

Figure S 18: Correlation of pKa_{exp} vs $V_{S,max}$. R^2 shown in each panel.
